# Bibliometric analysis of evolutionary trajectory and prospective directions of LAG-3 in cancer

**DOI:** 10.3389/fimmu.2024.1329775

**Published:** 2024-02-08

**Authors:** Jin Wang, Siying Wang, Yuting Zhang, Wei Zhang

**Affiliations:** Department of Breast Surgery, The First Affiliated Hospital, Jinan University, Guangzhou, China

**Keywords:** bibliometric analysis, LAG-3, cancer, immunotherapy, Citespace, VOSviewer

## Abstract

**Objectives:**

Perform a bibliometric analysis on the role of LAG-3 in the domain of cancer, elucidate the prevailing areas of research, and visually depict the evolutionary trajectory and prospective directions of LAG-3 research over the past twenty-three decades.

**Materials and methods:**

Between 2000 and 2023, a comprehensive review of scholarly articles pertaining to LAG-3 research in the context of cancer was carried out using the Web of Science Core Collection (WoSCC) database. Bibliometric analysis can be conducted by taking advantage of VOSviewer (version 1.6.16) and CiteSpace (version 6.2.R4). Create a network diagram to visually represent various authors, countries, and organizations while assessing the publishing years, journals, references, and keywords.

**Results:**

In conclusion, 1841 records were identified and published in 587 publications. These records were authored by 12,849 individuals affiliated with 2491 institutes across 74 countries. There has been a substantial surge in publications subsequent to 2013. The USA, China, and Germany gave the majority of records, amounting to 69.69%. American institutions actively engage in collaboration with institutions located in other countries. Triebel, F., Vignali, Dario A. A., Workman, Creg J. Drake, Charles G., and Elkord, Eyad are highly regarded authors in their respective fields. However, it is worth noting that Triebel exhibits limited collaboration with other writers. The examination of the role of LAG-3 in cancer and its potential for use in clinical settings is a discernible trend, as seen by keyword analysis.

**Conclusion:**

The scientific interest in and attention towards LAG-3 has experienced a significant rise since 2013. The United States is leading the way, with China following closely behind. Promoting collaboration among writers, nations, and institutions with varied backgrounds is imperative. The discipline of immunotherapy is currently seeing ongoing progress. A thorough investigation of the distinctive cis ligand TCR-CD3 complex of LAG-3 and its signal transduction mechanism is necessary. Additionally, it is worthwhile to explore novel combinations of LAG-3 therapy.

## 1 Introduction

Cancer contributes substantially to global mortality, accounting for approximately 10 million fatalities, or almost one-sixth of all deaths, in 2020 (1). By 2035, about 25% of the populace is projected to experience direct repercussions from cancer. This positive outcome can be attributed to the decline in smoking rates and developments in early detection and treatment of cancer (2). From 1991 to 2018, a consistent downward trend was observed in the cancer death rate, resulting in an overall reduction of 31% (3). The progression of cancer treatment commenced with the start of chemotherapy, surgery, radiation therapy, and immunotherapy, subsequently leading to the investigation and examination of gene therapy and nanomedicines (4, 5). In the past few years, cancer treatment has witnessed a significant rise in the prominence of immunotherapy, owing to the ongoing advancements in medical technology. It can provide effectiveness without toxicity and has the potential for lifelong immune memory to prevent recurrence (6). Hence, immunotherapy is anticipated to be included in numerous treatment regimens and is essential in treating diverse cancer types (7, 8).

The utilization of antibodies that inhibit the cytotoxic T lymphocyte antigen 4 (CTLA-4) (9) and programmed cell death protein 1 (PD-1) (10) in the treatment of cancer patients not only signifies the triumph of immune checkpoint blockade therapy but also signifies the initiation of a novel epoch in the field of cancer immunotherapy (7, 11). This confirms that inhibiting coreceptors is the key to immune cells not attacking tumor cells and tumor tissues. The application of specific blockade of coreceptors PD-1 and CTLA-4 in the context of cancer immunotherapy exhibits a substantial improvement in the prognostic outcomes for numerous populations of cancer patients, thereby significantly altering the landscape of cancer treatment (12–14). Nevertheless, despite the remarkable efficacy of CTLA-4 and PD1-PDL1(programmed cell death 1 ligand 1)-targeted therapies in cancer immunotherapy, a significant proportion of patients exhibit a restricted response to these treatments. Additionally, adverse events associated with immunization have been observed during the period of treatment (15–18). Consequently, there is an urgent demand for novel therapies that offer enhanced efficacy while minimizing toxicity.

Numerous activating and inhibitory coreceptors have been found besides PD-1 and CTLA-4 (19, 20). Lymphocyte activation gene 3 (LAG-3, CD223) emerges as a prominent target following PD-1 among the coreceptors mentioned above. LAG-3 is classified as a type I transmembrane protein, including four immunoglobulin-like domains designated as domain 1 (D1) through domain 4 (D4). The expression of LAG-3 is absent in naive T cells. However, it becomes expressed on CD4+ and CD8+ T cells upon antigen stimulation. Moreover, the degree of expression positively correlates with the immunosuppressive potency of LAG-3 (21, 22). CD4+ and CD8+ T lymphocytes exhibit sustained high expression of inhibitory coreceptors, particularly LAG-3, as a result of prolonged exposure to antigens such as viruses (23), bacteria (24), and parasita (25, 26). In addition, tumors also result in a state of t-cell dysfunction through extensive antigen exposure, depriving them of effective roles and functions, and such T cells are also referred to as exhausted T cells. The inhibition of LAG-3 has been demonstrated to effectively restore functionality in exhausted T cells and enhance the immune response against infections, albeit to a lesser degree compared to the blockade of PD-1 (27–29). Within the tumor microenvironment (TME), there is a notable upregulation of LAG-3 expression on the outer membrane of tumor-infiltrating lymphocytes (TIL) (30, 31). This high expression has the capacity to impede TIL proliferation and induce an arrest in the cell cycle, thereby suppressing the anti-tumor immune response mediated by T cells. Consequently, this process finally facilitates the evasion of the immune system by the tumor (32). The presence of LAG-3 has been detected in diverse cancer types, including breast cancer (33), melanoma (34), colorectal cancer (35), Hodgkin’s lymphoma (36), ovarian carcinoma (30), chronic lymphocytic leukemia (37), multiple myeloma (38), hepatocellular carcinoma (39), and gastric carcinoma (40). Over 30 types of anti-LAG-3 antagonists have progressed to the clinical development and trial phase for treating hematological tumors, breast cancer, renal cell carcinoma, melanoma, colon cancer, and other tumors (20). Nevertheless, our comprehension of LAG-3 in immunosuppression, signal transduction, epigenetics, ligands, etc., remains restricted, and the precise mechanism by which it interacts with other immunological checkpoints remains uncertain. What is the present state of combination therapy in clinical applications, including LAG-3 and PD-1 inhibitors? This bibliometric analysis aims to offer a comprehensive understanding of the development of LAG-3 in the cancer area by examining published records and performing a detailed analysis of the present research progress.

## 2 Research methods

The field of bibliometrics emerged as a distinct study in the year 1969 (41) and has since been extensively used to analyze literature (42). Its application has proven valuable in tracking the progress and trends of impactful publications. During the analytical process, performance analysis is undertaken to assess the progress of a particular field. This is achieved by gathering comprehensive data about several aspects, including authors, countries, institutions, keywords, journals, and references. In addition, use contemporary computer technology, images, and visual results to investigate the inherent relationships among this information for visual analysis. Ma and Xi (43) underscored the significance of employing visual analysis methods in Bibliometrics, as it aids in understanding data and enhances the comprehensiveness of the results.

**Figure 1:**
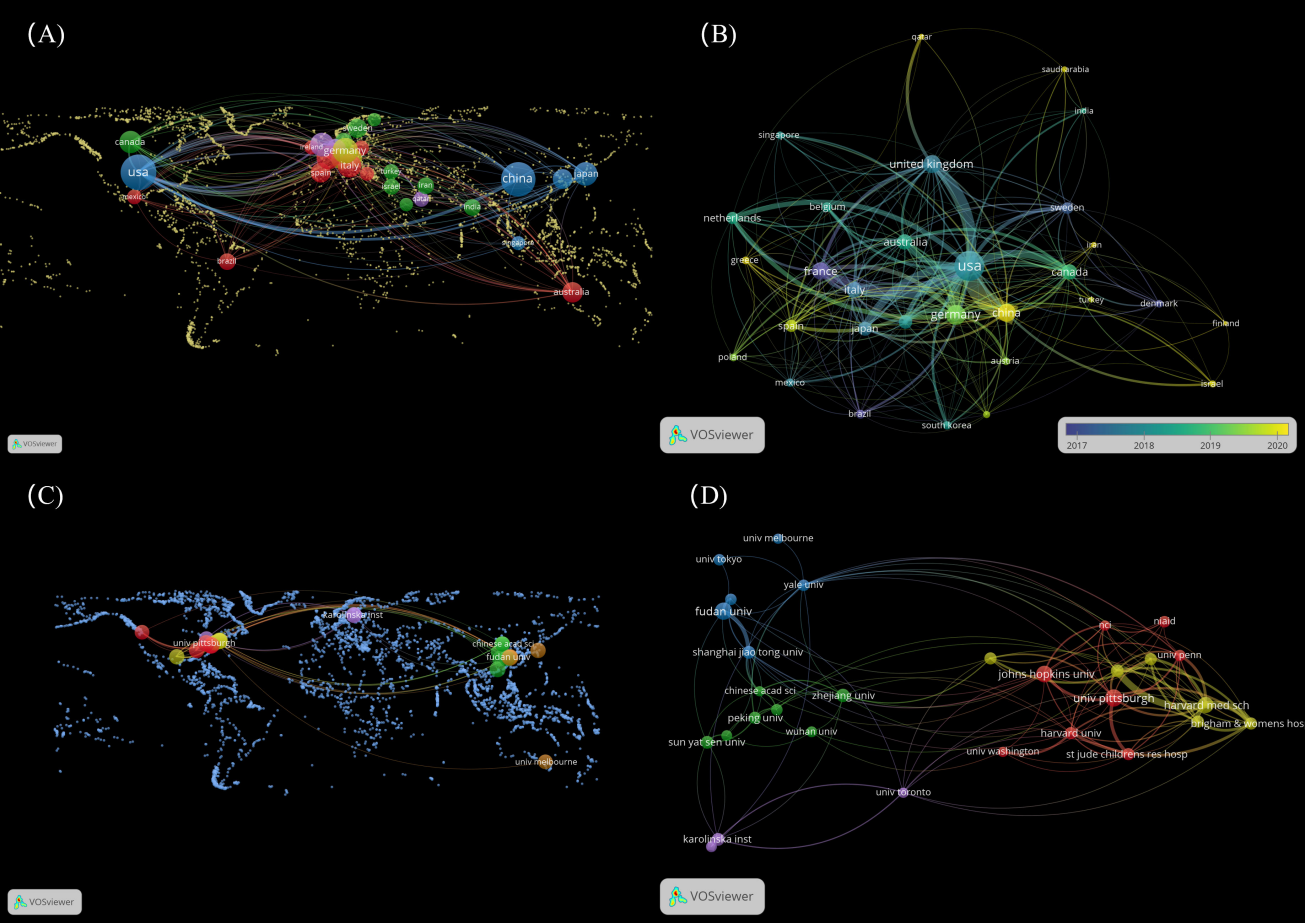
Distribution of countries and organizations. **(A)** The top 30 countries on the world map for LAG-3 research in cancer. **(B)** Dynamics and trends of countries over time (Top 30). **(C)** The top 30 organizations on the world map for LAG-3 research in cancer. **(D)** The relationships of the top 30 institutions are displayed on the map. (To generate panels **A, C**, utilizing the “MAP” file in VOSviewer is necessary. Additionally, one must retrieve nation and institution data and supplemental geographic boundary information through the web. The detailed operational procedure can be located in the **Supplementary Materials 1**).

**Figure 2:**
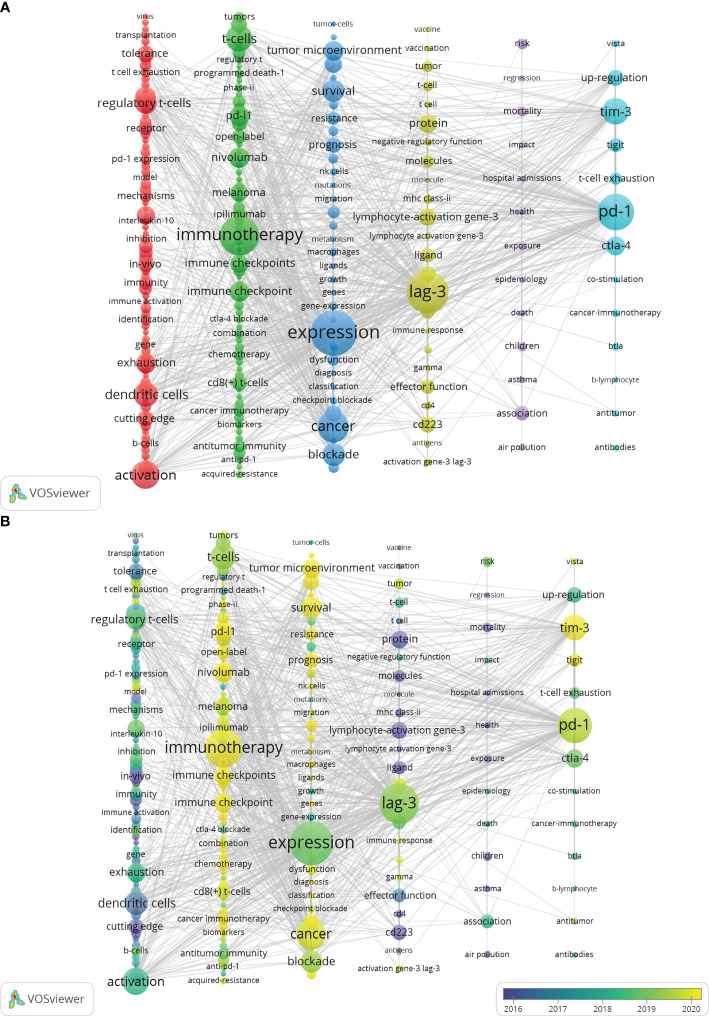
Distribution of keywords. **(A)** Network map of the frequency keywords (Top225). **(B)** Dynamics and trends of the frequency keywords over times (Top225). (To produce **Figure 2**, utilizing VOSviewer’s “MAP” file is necessary and must be employed in conjunction with Pajek. The detailed operational procedures can be found in the **Supplementary Materials 2**).

In this study, the software tools CiteSpace (version 6.2.R4) and VOSViewer (version 1.6.16) were employed to extract and analyze publication data, ultimately creating a knowledge graph. Microsoft Office Excel 2021 is used to process data and generate annual publication trend charts. Pajek (Version 5.17) is used for more advanced processing of scientific knowledge map generated by VOSViewer. In this article, **Figures 1A, C** and **Figure 2** are produced by VosView and Pajek. These software programs provide unique advantages and can effectively supplement one another. CiteSpace is a software application developed in Java that is utilized to analyze and visualize co-citation networks (44). The platform enables users to capture many temporal snapshots of a particular domain and combine them to obtain a comprehensive perspective of its chronological progression and temporal distribution. This facilitates a thorough comprehension of this field’s developmental trajectory and temporal patterns (45, 46). VOSviewer has outstanding characteristics, such as its simplistic graphical representation and aesthetically pleasing imagery (47). It offers a diverse range of visual perspectives, encompassing network, overlay, and density visualization. This study utilized Citespace to generate the dual-map overlay of journals, do burst analysis on keywords and references, and perform co-citation analysis on references. The VOSviewer software functions as a tool for doing co-citation analysis and subsequently visualizing the knowledge structure.

### 2.1 Data source and data analysis

**Figure 3:**
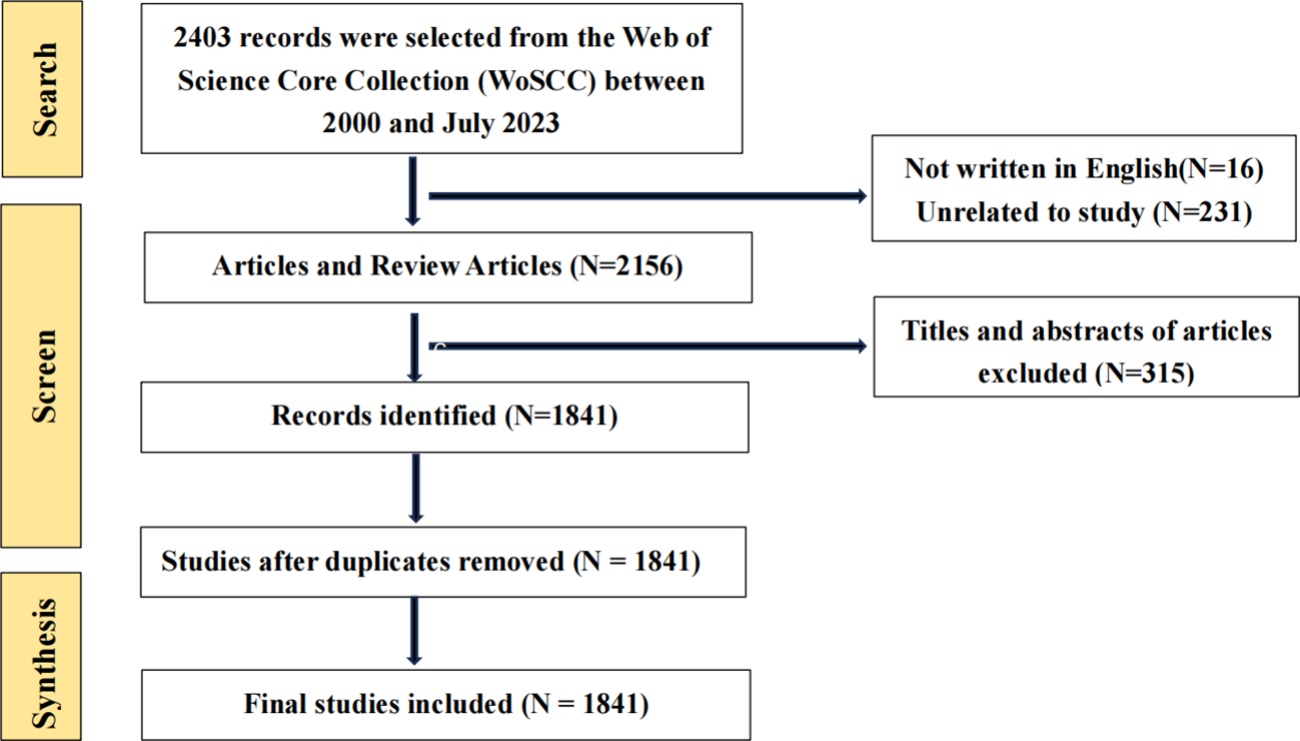
Process diagram of literature retrieval.

The data source for this study is the Web of Science Core Collection (WoSCC) database. The WoSCC database is widely regarded as a very reliable and comprehensive resource for conducting bibliometric analyses across diverse fields of study (48–52). The data collection for this study was conducted over a single day (July 10, 2023). The selected indexes for the study were the Social Sciences Citation Index (SSCI) and the Science Citation Index Expanded (SCI-Expanded). The chosen search strategy was TS= (“Lymphocyte activation gene 3” OR “lymphocyte-activation gene 3” OR “Lymphocyte activation gene-3”) OR TS= (LAG3 OR LAG-3 OR Lag3 OR “LAG-3 protein” OR “Lag3 protein”) OR TS= (CD223 OR CD223 Antigen*) AND TS= (cancer* OR tumor* OR Neoplasia*). With a period starting from 2000 to July 2023. The specified timeframe for completion is July 10, 2023. A total of 2403 references were retrieved via the search process. The literature was categorized as Articles and Review Articles, with English being the chosen language. Nevertheless, there are many problems brought by the literature directly through the search formula, such as duplication or inconsistency with the content of this study. Hence, before doing the analysis, we processed additional data to eliminate articles irrelevant to this study. To minimize the influence on the final results, we deleted papers that were not closely or distantly linked to this study based on carefully examining their titles and abstracts. After thoroughly reviewing each document, 1841 valid references were eventually acquired (**Figure 3**).

The data collection utilized in this study comprised 1841 documents, which were contributed by 12,849 writers affiliated with 2491 different organizations spanning 74 countries. Five hundred eighty-seven journals were used for publication, with 59,697 journal citations referenced.

**Figure 4:**
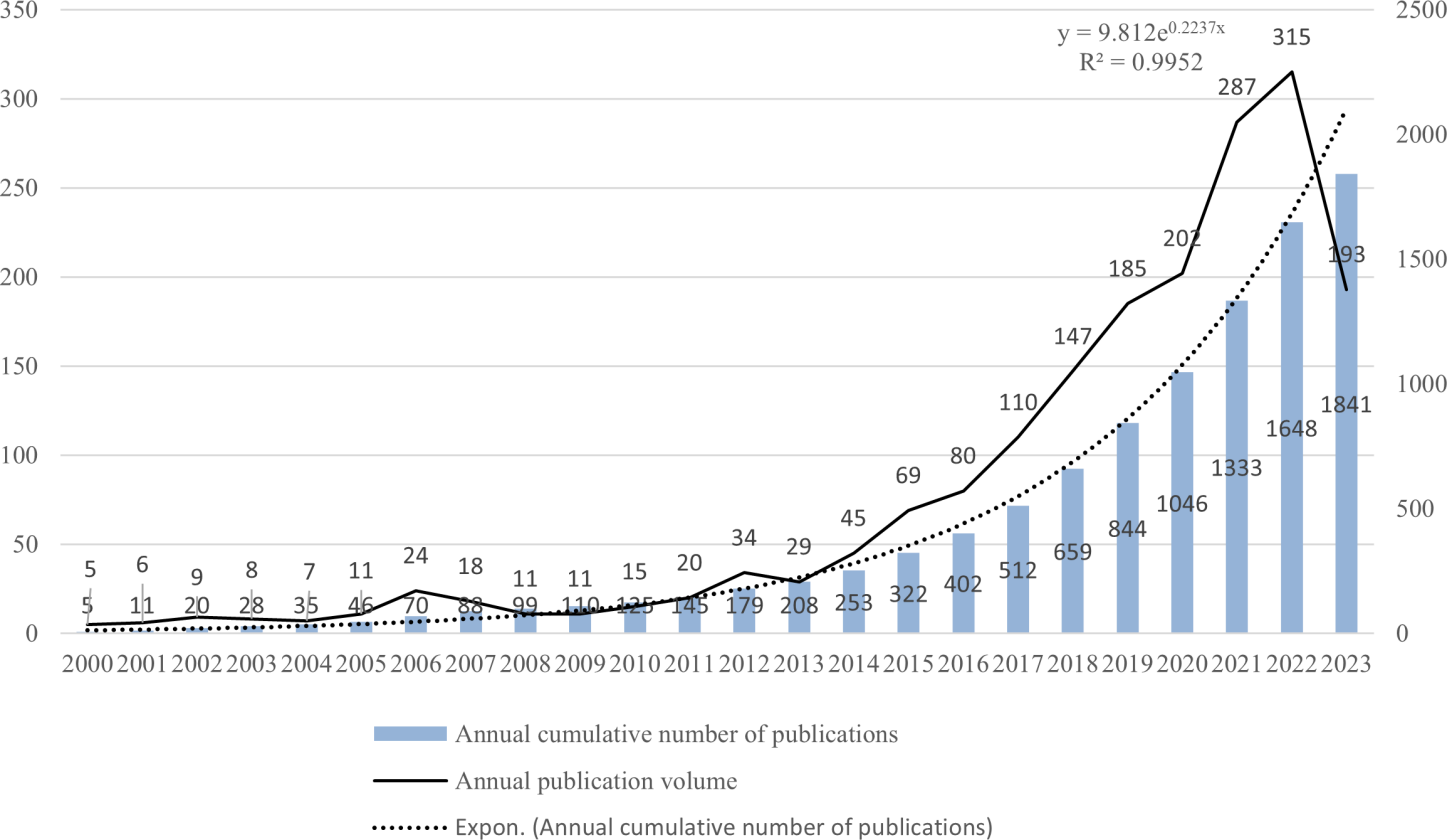
Global publication output and future publication trends of LAG-3 research in the field of cancer from 2000 to 2023.

**Figure 4** illustrates the worldwide publishing output and expected publication trends for LAG-3 research in the field of cancer, spanning the period from 2000 to 2023. The growth tendency can be categorized into two distinct stages. The initial phase lasted from 2000 to 2013, during which there was a marginal increase in the number of publications. However, the yearly academic results remained below 35. During the subsequent phase, there was a significant increase in the number of yearly publications, particularly beyond the year 2020. Between 2013 and 2023, the aggregate quantity of scholarly articles published throughout the previous ten years amounted to 1662, representing a proportion of 90.28% of all studies contained within the analysis. There is an anticipation that the number of publications throughout 2023 will persistently rise. (Note: The statistics about the annual publications in 2023 solely encompass those released up to July 10, 2023).

## 3 Descriptive statistics

### 3.1 Bibliometric analysis of authors

By comprehensively analyzing the published works of numerous authors, one can gain a deeper understanding of the prominent researchers and fundamental research trends within the area. The publication of 1841 papers yielded a total of 12,849 authors. **Table 1** presents a comprehensive overview of the Top 10 writers, encompassing their respective names, publications, and the average number of citations received in each publication. The group of authors ranked in the top 10 collectively generated a total of 166 papers, which accounts for around 9.02% of the overall number of papers.

**Table 1:** The preeminent authors in the field of cancer research pertaining to LAG-3.

Rank	Author	Documents, n%	Citations	Average Citation/Pub-lication
1	Triebel, F(France)	30(1.63%)	2006	66.87
2	Vignali, Dario A. A. (USA)	28(1.52%)	5084	181.57
3	Workman, Creg J. (USA)	18(0.98%)	3439	191.06
4	Drake, Charles G. (USA)	16(0.87%)	3755	234.69
5	Elkord, Eyad (UK)	14(0.76%)	675	48.21
6	Fujio, Keishi (Japan)	13(0.71%)	572	44
7	Okamura, Tomohisa(Japan))	13(0.71%)	572	44
8	Yamamoto, Kazuhiko (Japan)	13(0.71%)	572	44
9	Sumitomo, Shuji (Japan)	11(0.60%)	529	48.1
10	Kuchroo, Vijay K. (USA)	10(0.54%)	2252	225.2

Triebel, F He is the most prolific author, with a total of 30 publications, accounting for 1.63% of the total publications. His study primarily centers around EFTILAGIMOD ALPHA, a soluble LAG-3 protein. This protein is a major histocompatibility complex class II agonist, stimulating antigen-presenting cells. As a result, it leads to increased systemic type 1 T helper reactions and enhanced activation of cytotoxic CD8+T cells. Several clinical trials, specifically phase I or II, have recently investigated the combination of EFTILAGIMOD ALPHA (efti) with either the anti-PD-L1 antibody (pembrolizumab) or paclitaxel. These trials primarily focused on patients with head and neck squamous cell carcinoma (HNSCC), breast cancer, and non-small cell lung cancer (NSCLC) (53–56). A recent phase I clinical trial evaluated the efficacy and tolerability of combining left with nivolumab to treat advanced solid tumors. The results of this trial showed promising therapeutic effects and good tolerability. Further investigation and validation of this combination will be conducted in future phase II studies (57). Following closely after is Vignali, Dario A. A., with 28 publications (1.52%), Workman, Creg J. with 18 publications (0.98%), Drake, Charles G. with 16 publications (0.87%), and Elkord, Eyad with 14 publications (0.76%). The authors who ranked among the top 5 in average citation per publication are as follows: Drake, Charles G. secured the first position with an average of 234.69 citations per publication. Following Drake is Kuchroo, Vijay K., with an average of 225.2 citations per publication. Workman, Creg J. is ranked third with an average of 191.06 citations per publication. Vignali, Dario A. A. holds the fourth position with an average of 181.57 citations per publication. Lastly, Triebel, F is ranked fifth with an average of 66.87 citations per publication. Drake, Charles G. has made significant contributions to the field of LAG-3 research. The primary cancer types he researched are prostate cancer, renal cell carcinoma, advanced metastatic solid tumor, and bladder cancer (58–61). Nevertheless, based on the author’s recent publications, numerous clinical studies have focused on examining the combined effects of anti-PD-L1 with other treatments rather than concurrently treating different types of cancer with anti-LAG-3. This could be attributed to the fact that the research progress of LAG-3 in treating other tumors is still in its preliminary stages (59, 62). He engages in a tight collaboration with Vignali Dario A., with shared research interests in the fields of oncology and immunology. Vignali Dario A. specializes in researching the role of LAG-3 in immunological suppression and signal transduction. In 2022, Vignali and colleagues proved that the inhibitory receptor LAG-3 can disrupt TCR signaling and T cell activation by reducing the pH level of immunological synapses. This results in the detachment of the tyrosine kinase Lck from CD4 or CD8 co-receptors (63). This study has garnered significant scientific interest. Furthermore, in 2023, the author evaluated the ligand, signal transduction, therapeutic uses, and other facets of LAG-3 (64).

**Figure 5:**
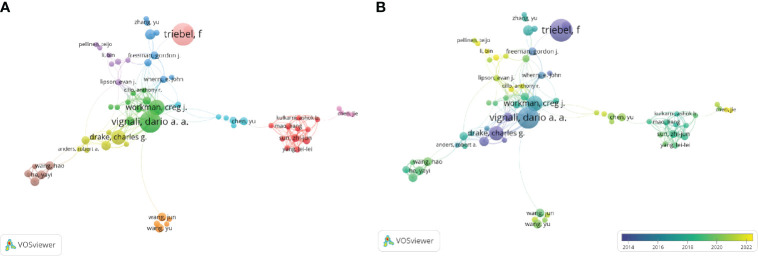
Distribution of authors. **(A)** A map showing the connections between the authors (Top181). **(B)** Dynamics and trends of authors over time (Top181).

By imposing a minimum publishing threshold for four articles and consolidating author synonyms using VOSviewer, the names “triebel, f.”and “triebel, Frederic.” were combined into “triebel, f.” A visual analysis was carried out on the top 181 authors, acquiring a collaborative network diagram depicting the authors (**Figure 5**). The top three writers showed a significant contribution to the field of cancer by publishing their work before 2016. Their vast knowledge repository serves as a valuable resource for advancing LAG-3 research. The largest cluster, indicated by the color red, comprised a total of 11 authors. This cluster exhibited strong potential as an emerging group, contributing to 51 (2.77%) publications. Notably, the majority of these publications were published after the year 2018.

### 3.2 Bibliometric analysis of journals

Dzikowski (65) posits that the influence of a journal is positively correlated with the number of papers published and the quantity of references cited. There is an upward trend between the quantity of impact a journal exerts, its publication capacity, and the number of references it cites. Hence, an analysis is conducted to examine and display the collected information, the number of publications, citations across all journals, and the average number of citations per publication.

**Table 2** lists the prominent ten journals focused on LAG-3 research within the cancer arena, arranged in descending order based on the frequency of publications. The five journals that have the highest number of articles are Frontiers in Immunology (135 publications), Journal of Immunology (63 publications), Cancers (52 publications), Journal for Immunotherapy of Cancer (43 publications), and Oncoimmunology (43 publications). This observation suggests that the five publications above maintain significant influence within the cancer research domain about LAG-3. According to the average citation per publication metric, Clinical Cancer Research ranks first with 90.34 citations per publication. Following closely is the Journal of Immunology, with 65.81 citations per publication. Oncoimmunology is ranked third with 29.63 citations per publication, while Plos One and Journal for Immunotherapy of Cancer have 26.03 and 23.23 citations per publication, respectively. This observation suggests an increasing level of interest in the field of LAG-3, as evidenced by the increasing popularity of the five mentioned publications. Clinical Cancer Research, a journal with 29 publications, achieved a position in the top 10 rankings, exhibiting the highest average number of citations per publication.

**Table 2:** The top 10 LAG-3 research journals published in the field of cancer research.

Rank	Source	Documents	Citations	Average Citation/Pub-lication	Quartile in category
1	Frontiers in Immunology (Switzerland)	135	2593	19.21	Q1
2	Journal of Immunology (USA)	63	4146	65.81	Q2
3	Cancers (Switzerland)	52	465	8.94	Q2
4	Journal for Immunotherapy of Cancer (USA)	43	999	23.23	Q1
5	Oncoimmunology (USA)	43	1274	29.63	Q1
6	Frontiers in Oncology (Switzerland)	40	387	9.68	Q2
7	Scientific Reports (England)	37	381	10.3	Q2
8	Plos One (USA)	36	937	26.03	Q2
9	Cancer Immunology Immunotherapy (USA)	29	470	16.21	Q1
10	Clinical Cancer Research (USA)	29	2620	90.34	Q1

### 3.3 Bibliometric analysis of countries and institutions

Synonyms from some nations, such as publications from Taiwan, have been consolidated with publications originating from China. Similarly, publications from England, Scotland, Northern Ireland, and Wales have been amalgamated into publications from the United Kingdom. A total of 2,491 institutions from 74 countries have together published 1,841 publications. The correlation between a country’s influence and its publication count, and the average number of citations per publication is evident. **Table 3** presents an overview of the 10 top countries engaged in cancer research about LAG-3. The five leading countries in terms of published articles are as follows: the United States of America (615 articles, accounting for 33.41% of the total), China (514 articles, representing 27.92% of the total), Germany (154 articles, comprising 8.37% of the total), Japan (114 articles, making up 6.19% of the total), and the United Kingdom (110 articles, constituting 5.98% of the total). It is worth noting that the remaining countries produced fewer than 110 articles each. Switzerland is positioned in tenth place in terms of the number of publications, yet it obtains the topmost position in average citation per publication, according to numerical values. China, which possesses the second-highest number of papers, is positioned at the 10th level regarding average citation per publication.

**Table 3:** TOP 10 countries had studies on LAG-3 published in the field of cancer.

Rank	Country	Documents,n%	Citations	Average Citation/Pub-lication
1	USA	615(33.41%)	36540	59.41
2	China	514(27.92%)	12144	23.63
3	Germany	154(8.37%)	5303	34.46
4	Japan	114(6.19%)	3961	34.75
5	United Kingdom	110(5.98%)	5253	47.75
6	France	108(5.87%)	5121	47.42
7	Italy	106(5.76%)	4039	38.1
8	Canada	84(4.56%)	3549	42.25
9	Australia	62(3.37%)	2623	42.31
10	Switzerland	58(3.15%)	3862	66.59

Set a minimum threshold of 12 publications and build a nationwide network map. According to the data presented in **Figures 1A, B**, the map consists of 30 nodes and 254 linkages. The dimensions of the nodes in the network diagram correspond to the number of national publications, while the quantity of links indicates the level of collaboration intensity. The 30 countries were organized into five distinct clusters and engaged in active cooperation both within and between these groups. We conducted a visualization of 30 countries on a global map, with most of them in Europe. It is worth mentioning that the nations with the most significant production in published works are North America (mainly the United States) and Asia (specifically China).

According to the data presented in **Table 4**, the leading organizations were derived from the United States and China, collectively producing 619 articles, which accounts for 33.62% of the total. The University of Pittsburgh emerged as the leading institution among the top 5, with a publication count 46 (representing 2.5% of the total). Fudan University secured the second position with 44 publications (2.39%), followed by Johns Hopkins University with 43 publications (2.34%). Harvard University and Zhejiang University ranked fourth and fifth, respectively, with 40 publications (2.17%) and 27 (1.47%). In the network diagram of the institution, nodes with a frequency exceeding 16 were observed to be 30 in number, while the links connecting these nodes amounted to 126. The 30 universities were grouped into five groups, as depicted in **Figure 1D**. By reviewing the data shown in **Figure 1C**; **Table 4**, it becomes evident that most of the 30 institutions under analysis are in the United States (15) and China (10). The remaining institutions are distributed among Sweden (2), Japan (1), Canada (1), and Australia (1), respectively.

**Table 4:** The top 30 organizations that published LAG-3 research in the field of cancer.

Rank	Organization	Country	Documents, n%	Citations	Average Citation/Pub-lication
1	Univ Pittsburgh	USA	46(2.50%)	2745	59.67
2	Fudan Univ	China	44(2.39%)	799	18.16
3	Johns Hopkins Univ	USA	43(2.34%)	5608	130.42
4	Harvard Med Sch	USA	40(2.17%)	1306	32.65
5	ZheJiang Univ	China	27(1.47%)	1288	47.7
6	Karolinska Inst	Sweden	26(1.41%)	761	29.27
7	Sun Yat Sen Univ	China	26(1.41%)	475	18.27
8	Shanghai Jiao Tong Univ	China	25(1.36%)	794	31.76
9	Massachusetts Gen Hosp	USA	25(1.36%)	745	29.8
10	Univ Texas Md Anderson Canc Ctr	USA	23(1.25%)	1051	45.7
11	Peking Univ	China	23(1.25%)	945	41.09
12	St Jude Childrens Res Hosp	USA	22(1.20%)	5931	269.6
13	Dana Farber Canc Inst	USA	22(1.20%)	3268	148.55
14	Harvard Univ	USA	22(1.20%)	2887	131.23
15	Brigham & Womens Hosp	USA	22(1.20%)	2374	107.91
16	Mem Sloan Kettering Canc Ctr	USA	22(1.20%)	2007	91.23
17	Univ Penn	USA	21(1.14%)	2310	110
18	Univ Tokyo	Japan	21(1.14%)	880	41.9
19	Yale Univ	USA	20(1.09%)	1999	99.95
20	NIAID	USA	20(1.09%)	1265	63.25
21	TongJi Univ	China	20(1.09%)	532	26.6
22	Capital Med Univ	China	20(1.09%)	508	25.4
23	ZhengZhou Univ	China	19(1.03%)	837	44.05
24	Karolinska Univ Hosp	Sweden	18(0.98%)	518	28.78
25	WuHan Univ	China	18(0.98%)	416	28.11
26	NCI	USA	17(0.92%)	1156	68
27	Univ Melbourne	Australia	17(0.92%)	989	58.18
28	Univ Toronto	Canada	17(0.92%)	548	32.24
29	Univ Washington	USA	16(0.87%)	917	57.31
30	Chinese Acad Sci	China	16(0.87%)	438	27.37

### 3.4 Co-citation and citation burst reference analysis

**Figure 6:**
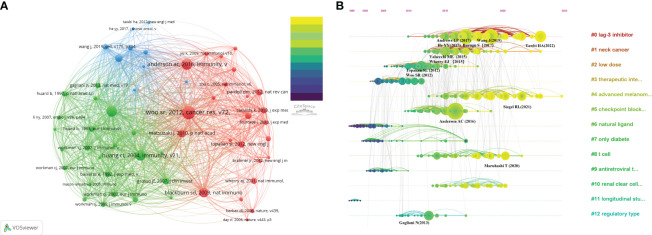
**(A)** Co-citation of cited references. **(B)** Timeline map of co-citation reference clustering.

The threshold number was set to 15 times, and VOSviewer software was applied to generate a co-citation map of literature references. A total of 56 references were obtained for subsequent co-citation analysis. **Table 5** displays the assembled list of the ten most frequently co-cited references about the investigation of LAG-3 in cancer research. The range of co-citations varied between 157 and 325. One reference (32) was referred to on more than 300 times, while four other references were cited between 200 and 300 times (22, 66–68). Additionally, five references were cited between 150 and 200 times (30, 69–72). To comprehensively comprehend the citation structure in the LAG-3 study, one ought to set up a threshold of 15 occurrences and utilize VOSviewer software to generate a co-citation map of the literature references. This approach will yield 56 references for subsequent co-citation analysis of the cited references. According to the depiction in **Figure 6**, the co-citation network of highly co-cited publications exhibits an apparent division into three primary clusters, each corresponding to a distinct color as depicted in the picture. Clusters with a green hue tend to recognize and explore the LAG-3 molecule in the context of infection and immune responses. The majority of academic articles in the green cluster were written before 2005. The discovery of LAG-3 can be attributed to Triebel (22) and their colleagues, as evidenced by the earliest article on the subject. Red clusters further investigate the functions of LAG-3 in autoimmunity, tumor immunity, and targeted immunity, with most articles published from 2005 to 2015. The blue cluster is a gathering of academic research on immune targeting and checkpoint inhibitors, with most of the papers being published after 2015.

**Table 5:** Top 10 co-cited references related to LAG-3 research in the cancer field.

Rank	Co-cited reference	Co-cation
1	Woo Sr, 2012, CANCER RES, v72, p917 (32)	325
2	Anderson Ac, 2016, IMMUNITY, v44, p989 (66)	268
3	Huang Ct, 2004, IMMUNITY, v21, p503 (67)	262
4	Triebel F, 1990, J EXP MED, v171, p1393 (22)	256
5	Blackburn Sd, 2009, NAT IMMUNOL, v10, p29 (68)	228
6	Matsuzaki J, 2010, P NATL ACAD SCI USA, v107, p7875 (30)	186
7	Andrews Lp, 2017, IMMUNOL REV, v276, p80 (69)	183
8	Pardoll Dm, 2012, NAT REV CANCER, v12, p252 (70)	160
9	Grosso Jf, 2007, J CLIN INVEST, v117, p3383 (71)	159
10	Topalian Sl, 2012, NEW ENGL J MED, v366, p2443 (72)	157

In addition, we conducted literature co-citation analysis using CiteSpace and produced a clustered representation of reference co-citation analysis, as depicted in **Figure 6B**. The horizontal timeline illustrates the development and pattern of this cluster, while the vertical view visually compares the frequency of events within a specific period. The magnitude of the nodes corresponds to the influence of the literature. The LAG-3 inhibitor (#0) exhibits the most significant cluster, followed by neck cancer (#1) and low dose (#2). The current trending themes include LAG-3 inhibitor (#0), neck cancer (#1), advanced melanoma (#4), T cell (#8), and renal clear cell carcinoma (#10), and it is expected that they will remain popular in the future.

**Figure 7:**
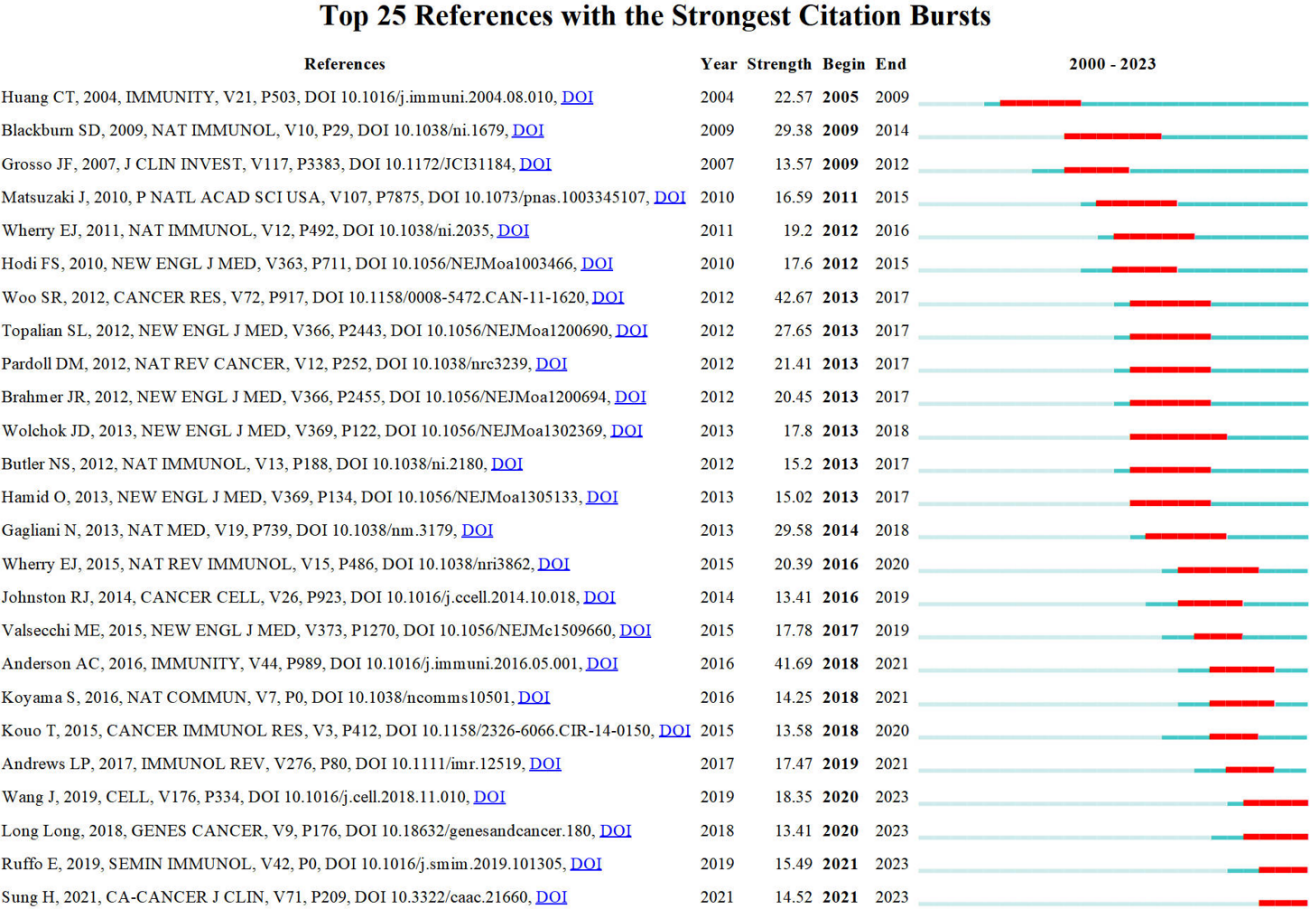
Top 10 co-cited references related to LAG-3 research in the cancer field.

Price (73) identified that scientific literature has two separate citation half-lives, each with unique characteristics. Notably, classic papers consistently demonstrate a high citation rate. On the contrary, the citation rate of transient articles experiences a rapid surge within a limited timeframe. Understanding the significance of transient articles at that time is crucial for developing cutting-edge fields in various disciplines. **Figure 7** displays the top 25 references that exhibit the strongest citation bursts. The first reference (67) with a citation burst appeared in 2005, most of which appeared between 2013 and 2017. Only four references (74–77) appear with a burst in the last three years.

### 3.5 Co−citation analysis on cited journals

**Figure 8:**
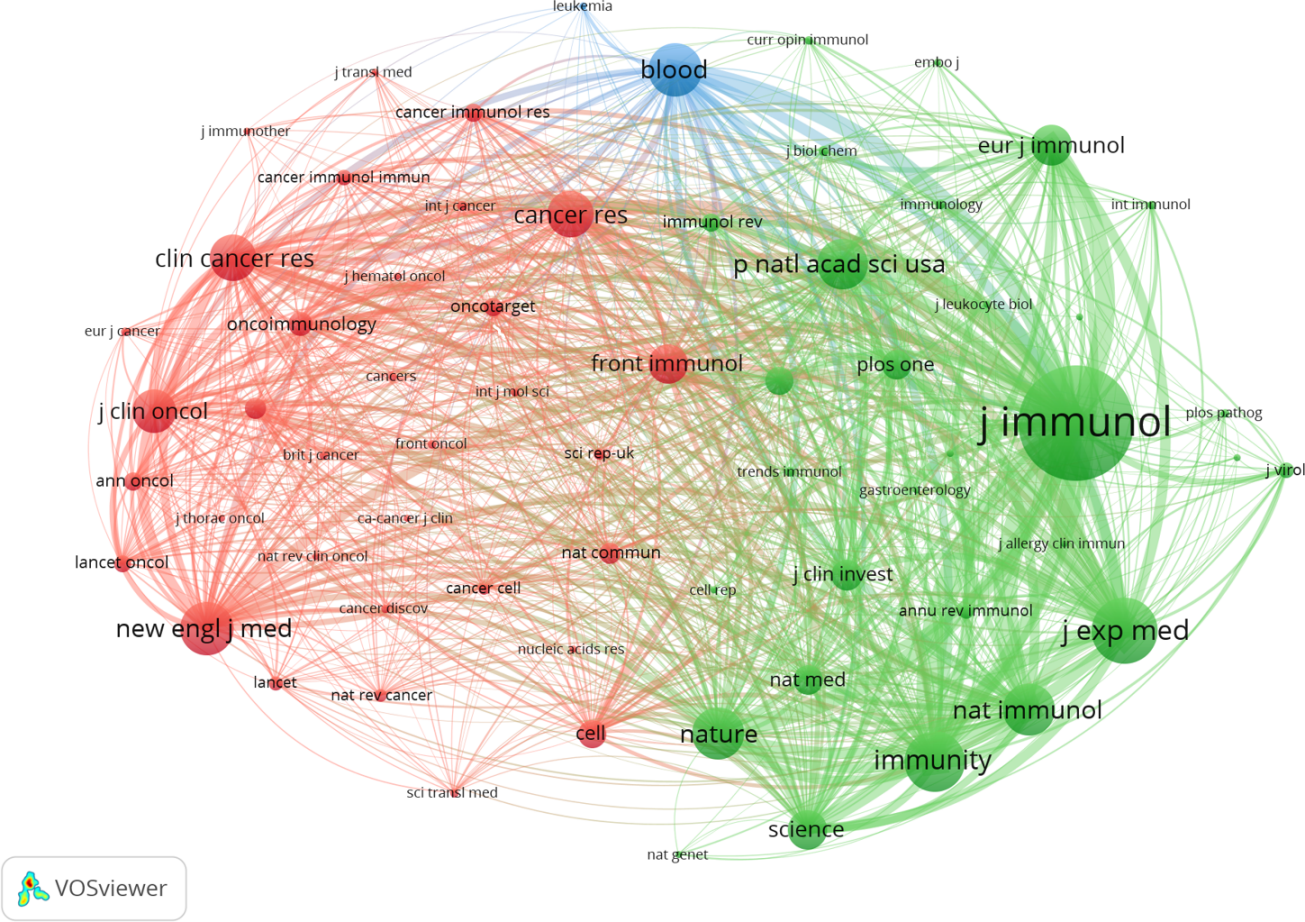
Co-citation of cited journals.

In the same way, by setting a minimum citation criterion of 260 for cited journals, we identified 65 journals to conduct a co-citation analysis. This research finally resulted in constructing a network graph comprising three distinct clusters, namely the immunological, cancer, and hematological systems (refer to **Figure 8**). According to the data presented in **Table 6**, the three most often cited journals are The Journal of Immunology (with 5984 citations), Journal of Experimental Medicine (with 3336 citations), and Immunity (with 3009 citations). Notably, two of the three publications, excluding the immunology journal from Q2, belong to the Q1 category.

**Table 6:** The top 10 co-cited journals of LAG-3 research in the cancer field.

Rank	Source	Citations	Quartile in category	Country
1	J Immunol	5984	Q2	USA
2	J Exp Med	3336	Q1	USA
3	Immunity	3009	Q1	USA
4	Blood	2631	Q1	USA
5	New Engl J Med	2616	Q1	USA
6	Nature	2582	Q1	England
7	Nat Immunol	2566	Q1	USA
8	P Natl Acad Sci Usa	2513	Q1	USA
9	Clin Cancer Res	2303	Q1	USA
10	Cancer Res	2291	Q1	USA

**Figure 9:**
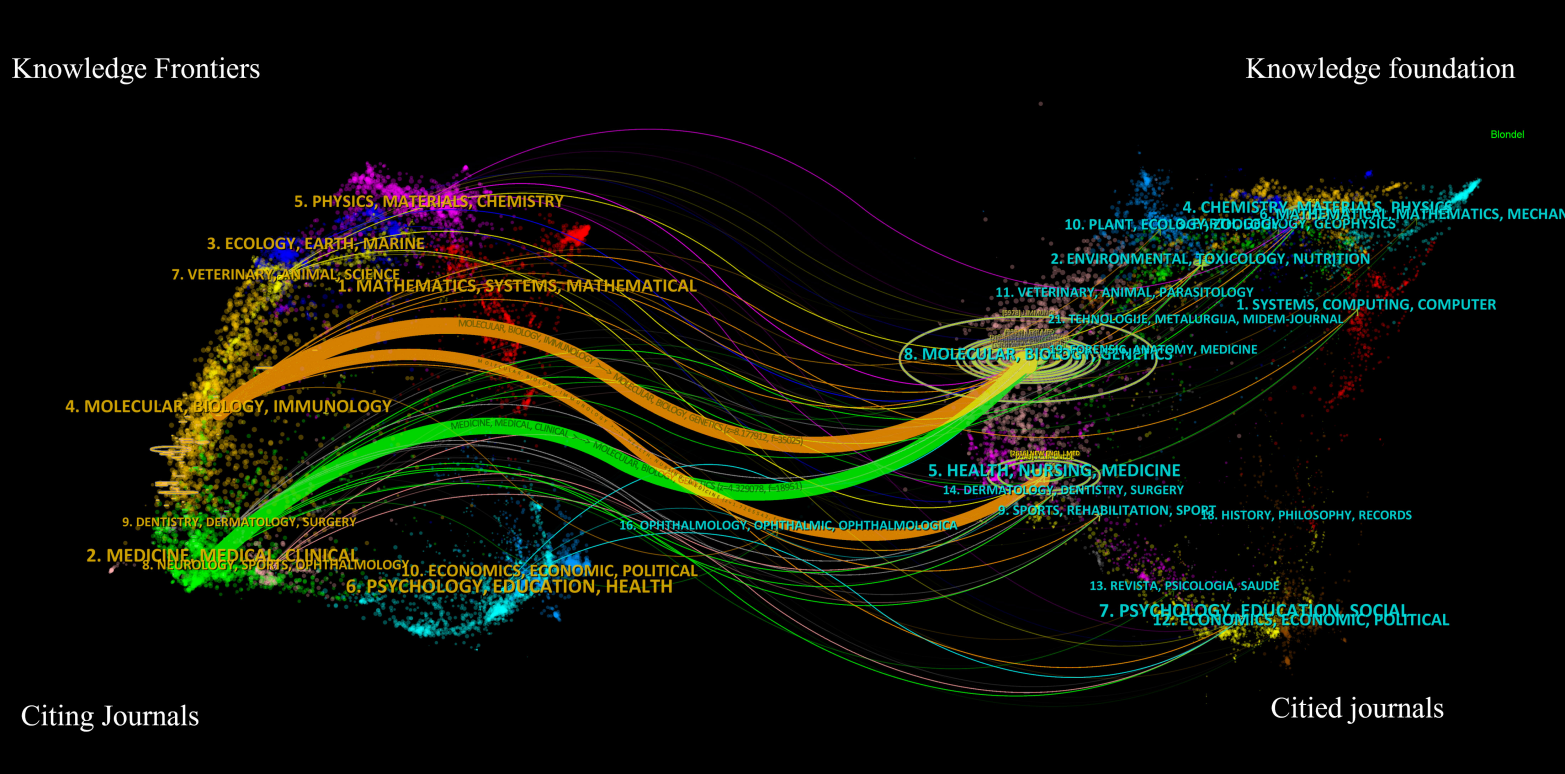
The dual-map overlay of journals related to LAG-3 research in the cancer field.

The dual-map overlay of journals is depicted in **Figure 9**. A visual representation of citing journals that represent cutting-edge knowledge is shown on the left side of the figure. In contrast, the right side visualizes cited journals that form the fundamental basis of knowledge. The label acts as a representation of the diverse study topics encompassed by the publication. The reference path is depicted by the colored curve, wherein each path originates at the map of citing journals and terminates at the map of the cited journals. A correlation exists between the width of the color curves and the frequency of citations. The existing map displays three main reference pathways. This suggests that scholarly articles published in journals specializing in Health, Nursing, Medicine, Molecular Biology, and Genetics are frequently cited and published in journals focusing on Molecular Biology and Immunology. Likewise, scholarly investigations in Molecular Biology and Genetics are referenced and disseminated in journals about Medicine, Medical, and Clinical areas.

### 3.6 Analysis of keywords

A complete collection of 6234 keywords was derived from a set of 1841 articles. Merely 3.6% of these keywords occur more than 14 times, while the large majority, approximately 69.35%, appear only once. This significant difference suggests that only a small percentage of keywords are used with high frequency. Six distinct clusters were formed after conducting a clustering analysis on the network map of the top 225 commonly used keywords (refer to **Figure 2A**). It is worth noting that “Expression,” “LAG-3,” and “Immunotherapy” were the three most significant nodes in the network. Furthermore, **Figure 2B** presents the overlay visualization depicting the top 225 frequency keywords spanning 2000 to 2023. The red clusters comprised a total of 75 keywords, which are possibly associated with immunological mechanisms, especially activation, dendritic cells, regulatory T-cells, exhaustion, and tolerance. The Green cluster contained a total of 66 keywords. The most prominent node within the Green cluster was identified as immunotherapy, which is well recognized as a focal priority in contemporary tumor therapy. Notably, this node encompasses several aspects of immunotherapy, such as immunological checkpoints, T cells, PD-L1, and nivolumab. The clusters characterized by a blue color exhibit a prominent node labeled “Expression,” which perhaps pertains to tumor therapy. Notably, keywords such as tumor microenvironment, blockade, survival, prognosis, and especially checkpoint blockade warrant increased attention within these clusters. The yellow clusters contain 24 keywords primarily associated with LAG-3 signaling mechanism. These keywords include lymphocyte-activation gene-3, LAG-3, protein, ligand, and molecules. The Purple Cluster comprised 13 keywords: association, health, risk, and mortality. The cyan cluster consisted of 13 keywords, including TIM-3, CTLA-4, and PD-1, which exhibit comparable functionalities to LAG-3 and hold significant importance in autoimmune, tumor-immune, and anti-infective immune responses.

**Figure 10:**
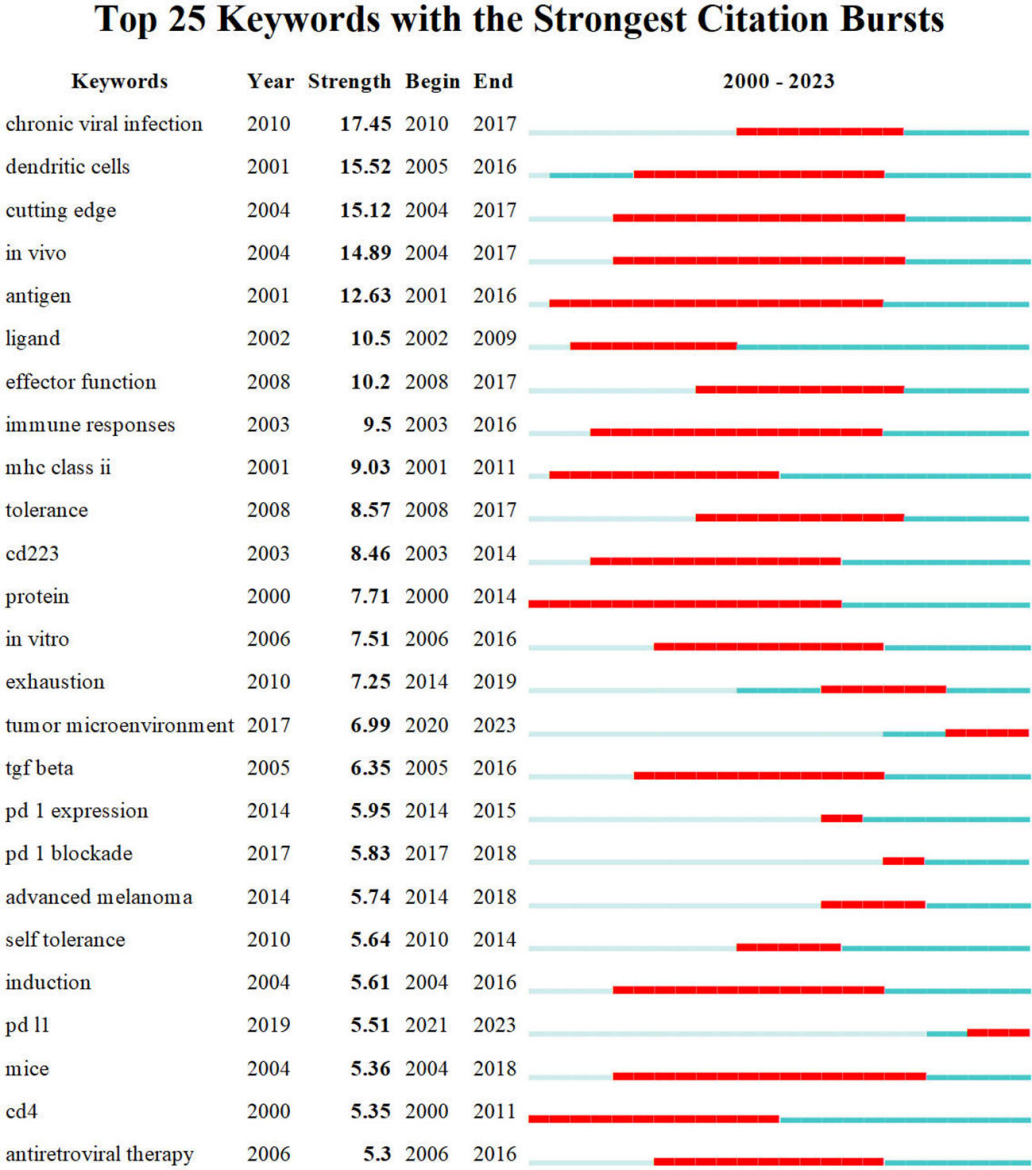
Top 25 keywords with strongest citation bursts.

Additionally, results depicted in **Figure 2B** indicate that the most prominent nodes, namely “Expression,” “LAG-3,” and “Blockade,” emerged predominantly in the year 2019. Following this, there has been a progressive rise in the occurrence of several significant keywords, such as “Immunotherapy,” “TIM-3,” “PD-1,” “PD-L1,” and “Cancer” after the year 2019. This surge in interest has been observed to grow significantly in subsequent periods. According to the data displayed in **Figure 10**, it can be observed that all keywords exhibiting citation bursts had their initial appearance in the year 2000. The study of antigens garnered significant attention in the field of research between 2001 and 2016. The surge in citations for the TME and PD-L1 was most pronounced in 2020. The time interval during which the most significant citation bursts for the keyword CD223 occurred spanned from 2003 to 2014.

**Table 7** presents a comprehensive summary of the frequency of the top 20 keywords. Keywords with a higher frequency exhibit a greater level of popularity on LAG-3 within the field of cancer research. Notably, the words “Expression,” “Immunotherapy,” “Activation,” and “Blockade” are among the keywords that maintain a significant presence. Moreover, the TME and immunological checkpoints have emerged as prominent subjects of interest in oncology. TIM-3, a second-generation immune checkpoint inhibitor, shares similar activities with LAG-3. It is observed more frequently than CTLA-4 and PD-L1 and is likely to become a prominent area of research following LAG-3. Ultimately, by a comparison of the frequency of keyword occurrences, we have determined that terms such as “regulatory T cells” and “dendritic cells (DC)” have closely followed “T cells.” This also suggests that researchers investigating the immunosuppressive mechanism of LAG-3 have progressively broadened their research scope beyond T cells, specifically CD8+T cells, to encompass other kinds of cells in the TME.

**Table 7:** The top 20 keywords in terms of frequency for LAG-3 research in the cancer field.

Rank	Keyword	Occurrences	Rank	Keyword	Occurrences
1	Expression	478	11	Survival	138
2	LAG-3	410	12	Regulatory T-cells	134
3	Immunotherapy	371	13	Responses	127
4	PD-1	333	14	Nivolumab	125
5	Cancer	249	15	PD-L1	119
6	Activation	210	16	Immune Checkpoint	117
7	T-cells	193	17	Tumor Microenvironment	117
8	Tim-3	188	18	Exhaustion	114
9	Dendritic Cells	163	19	CTLA-4	108
10	Blockade	144	20	Protein	103

## 4 Discussion

### 4.1 General information

In this study, we collected articles from the WoSCC database that explored LAG-3 research in cancer. In total, 1841 references were included and analyzed, which were published in 587 journals. Twelve thousand eight hundred forty-nine authors from 2491 institutions in over 74 nations contributed to these references. Numerous studies were published in American journals, and these papers were often cited simultaneously. Eight of the top 10 co-cited journals, categorized as Q1 in **Table 6**, were published in the United States. It indicates that recognized publications are well-liked all across the world. Four of the top 10 authors are from the United States, four are from Japan, and one each is from the United Kingdom and France. The average number of citations per publication for four American authors is high, yet it is similar among the four Japanese authors.

Authors from The United States work closely with authors from different countries, according to the network map of authors. In contrast, Triebel, F., from the UK, cooperates less frequently with authors from other countries. The top 3 countries, which accounted for 69.69% of all articles, were the USA, China, and Germany. The United States was far ahead of the other top 10 countries since it provides 33.41% of all articles and has the second-highest average number of citations per publication (behind Switzerland) among the top 10. This shows that American researchers dominate this area of study. In terms of institutions, half of the top 30 institutions were from the United States, and they contributed 381 publications, or 20.7% of the total number of published articles, to LAG-3 research on cancer. While combined with analysis of countries and institutions, American organizations cooperated more closely with organizations globally, while Chinese organizations collaborated more closely with domestic organizations and fewer with international organizations. Despite having the second-highest volume of publications, China needs more research capabilities in this area, as reflected by the low average publication citation rate. One possible explanation for this phenomenon could be China’s high pace of technological and scientific advancements, where the sole nation in development is in the top ten list. This is also the reason why China’s research on LAG-3 in the field of oncology is insufficient compared to other countries.

The literature searched primarily consists of publications after 2000, as a result of utilizing the SSCI and SCI-Extended search indexes. Hence, our primary focus lies in analyzing the publication trend after 2000. The initial publication on LAG-3 in the field of cancer was documented in 1990, and the average yearly count of articles between 2000 and 2004 was below 10. Between 2004 and 2013, there was a gradual increase in published articles; nevertheless, the yearly academic output remained beneath 35 papers, with a mere 29 articles being published in 2013. Following 2013, there was a notable increase in the yearly production of academic content. In the period spanning from 2013 to 2023, a comprehensive analysis encompassing an overall 1,662 scholarly articles about LAG-3 was conducted. These papers accounted for roughly 90.28% of the literature analyzed in the study. The likely attribution of the increase in the general number of articles after 2013 encompasses numerous noteworthy aspects. Firstly, in 2013, Science Magazine designated cancer immunotherapy as the yearly breakthrough (78). Simultaneously, there has been a significant surge in academic articles PD-1 and CTLA-4 in cancer research (79, 80). Consequently, LAG-3, as one of the auxiliary receptors involved in immune inhibition, has garnered considerable attention from researchers worldwide. Secondly, the growing authorization of PD-1 and PD-L1 inhibitors for treating diverse tumor types (81, 82) has garnered significant attention to the combined utilization and effectiveness of anti-LAG-3 medicines and anti-PD-1 agents. Over ten medications are being investigated in clinical trials for their efficacy in treating various malignancies by targeting LAG-3. These drugs are being studied individually and in combination with anti-PD-1 or anti-PD-L1 inhibiting antibodies (14). Finally, the third inhibitory receptor (IR), LAG-3, has been the focus of clinical research with antagonistic monoclonal antibodies (mAb). In 2013, Phase I clinical trials were initiated for BMS-986016 (69), a particular antagonist targeting LAG-3. The number of patents about LAG-3 and its link with cancer has grown substantially since 2014 (20), hence establishing LAG-3 as a burgeoning focal point for cancer treatment.

### 4.2 The analysis of transient articles

The influence of a co-cited publication is directly proportional to the number of citations it gets. The analysis of transient literature can reflect the cutting-edge topics of a certain period. By integrating the data provided in **Table 5** and **Figure 7**, it can be seen that nine publications (30, 32, 66–72) were identified as being among the top 10 co-cited references and also ranked within the top 25 for strongest citation bursts. One article (22) failed to meet the inclusion requirements and could not make it into the top 25 references with the strongest citation bursts. This indicates that detecting the top 10 co-cited references displaying citation bursts at specific times can indicate research fields currently receiving significant attention. The first occurrence of the strong bursts appeared in an academic paper published in 2004, and the effects persisted from 2005 to 2009 (67). The present paper argues that the membrane localization of LAG-3 acts as a unique biomarker for induced regulatory T cells (Tregs). It further suggests that LAG-3 is involved in the modulation of Tregs’ suppressive function, hence contributing to the regulation of their activity.

In 2009, another strong burst occurred due to a research article released by Blackburn et al. (68) in the same year. The article points out that various inhibitory receptors in CD8+ T cells create a negative regulatory mechanism essential in treating chronic infections and possible therapeutic interventions. The most prominent boost in citations in 2013 can be attributed to a research article by Wolchok et al. (83) published in the same year. The present study conducted a phase 1 clinical trial to assess the effectiveness and security of the combined treatment of nivolumab and ipilimumab in a cohort of individuals diagnosed with advanced melanomas. The study confirmed that the utilization of a combination therapy integrating nivolumab and ipilimumab exhibits manageable safety profiles while also showing significant instances of rapid and significant tumor regression among numerous patients.

Over the previous five years, strong bursts were observed in two scholarly articles published in 2018 and 2019. In 2018, Long et al. (74) performed a study that comprehensively understood the crucial role of LAG-3 as an immune checkpoint inside the tumor’s microenvironment. The study’s primary objective was to investigate the intricate mechanisms underlying the connections between LAG-3 and other immunological checkpoints, with a particular emphasis on PD-1. Additionally, the study summarized the recent advancements made in LAG-3 targeted immunotherapy. In 2019, Wang et al. (77) published research indicating that the FGL1 protein, predominantly released by hepatocytes, serves as the principal immunosuppressive ligand of LAG-3 in the context of normal physiological conditions. The study’s findings demonstrate the existence of an immune evasion mechanism known as the FGL1-LAG-3 pathway. Furthermore, the authors propose that the FGL1-LAG-3 pathway could be a promising target for immunotherapy against cancer.

### 4.3 Major research directions, hotspots and evolutionary trajectories of LAG-3 in oncology

Following its discovery by Triebel et al. in 1990 (22), the number of studies focusing on LAG-3 has steadily grown. In contrast to PD-1 and CTLA-4, it has been determined that the loss of LAG-3 alone does not lead to the development of autoimmunity in non-autoimmune-prone mice. However, when LAG-3 is either absent or blocked, there is a significant increase in the onset of autoimmune diabetes in non-obese diabetic (NOD) mice, with a 100% incidence rate (84). Deadly autoimmune myocarditis is observed in mice with a combined deficit of LAG-3 and PD-1 (21). The elimination of LAG-3+ T cells in primates can potentially inhibit the development of delayed hypersensitivity reactions (85). In addition, some clinical studies using immune checkpoint inhibitors (ICIs) specifically targeting PD-1 and CTLA-4 have observed some immune related adverse events during the treatment process (irAEs) (86, 87). According to a meta-analysis conducted in 2018 (88), colitis was the predominant cause of irAE mortality among a cohort of 193 patients who received anti-CTLA-4 antibodies, with a mortality rate of 70%. Among the cohort of 333 patients that underwent treatment with anti-PD-1 or anti-PD-L1 antibody therapy, 115 fatalities were ascribed to pneumonia, accounting for 35% of the cases. Additionally, 75 deaths were classified as hepatitis, representing 22% of the cases, while 50 cases were linked to neurotoxicity function, representing 15%.

Given the present state of immunotherapy, developing a novel treatment approach that offers improved effectiveness and decreased toxicity is imperative. LAG-3 has been identified as an up-and-coming candidate for immunotherapy for cancer and is further substantiated by extensive studies conducted on models of animals. Therefore, conducting an in-depth analysis of the research on LAG-3 in tumors is necessary. Keywords are the fundamental elements extracted from an article, and evaluating keywords can reveal the current areas of research interest. We conducted cluster analysis on 225 frequently used keywords using VOSviewer, as depicted in **Figure 2**. Out of the six distinct clusters formed, the nodes identified as “expression,” “LAG-3”, and “immunotherapy” have the most significant level of importance. The forthcoming discourse will center on the topics of “LAG-3” and “expression,” and further illustrations of “immunotherapy” will be shown in the following section. In general, our comprehension of LAG-3 is steadily growing.

LAG-3 expression is controlled via several mechanisms, such as epigenetic, transcriptional, post-transcriptional, and post-translational processes (89). In recent years, more researchers have conducted the study mentioned above (90). Epigenetics is a vital factor in the development of cancer. During the process of carcinogenesis, the epigenome experiences many alterations, including global adjustments in histone patterns, complete removal of DNA methylation, and localized increase in methylation (91). In contrast to normal tissues, malignancies exhibit widespread hypomethylation of LAG-3, and the degree of methylation of the LAG-3 promoter is inversely associated with the expression level of LAG-3 messenger RNA (mRNA), which affects immune cell infiltration. Furthermore, the methylation status of LAG-3 is linked to the presence of immune cells (CD4+/CD8+T cells), interferon γ (IFN-γ) signaling (92, 93). The research above suggests that there may be a correlation between LAG-3 methylation levels and prediction and prognosis. Furthermore, this finding indicates that LAG-3 methylation could be a promising target for interventions in cancer treatment. Several transcriptional regulators linked to LAG-3 at the gene expression level have been identified. These include thymocyte selection-associated high-mobility-box proteins (TOX), members of the activated T-cell family of nuclear factors, and transcription factors of nuclear receptor subfamily four and group a, all of which contribute to increased expression of LAG-3 (94–96), which tends to lead to tumor progression and a poor prognosis. Following translation, lacking stimulation, most LAG-3 is not transported to the cell surface but instead undergoes degradation in the lysosomal compartment. However, upon stimulation, the movement of LAG-3 from the lysosomal box to the cell surface relies on specific structural domains in the cytoplasm of activated T cells, which signal through protein kinase C (PKC) (97). Further investigation is required to fully understand the precise mechanism by which LAG-3 is transported to the cellular membrane.

LAG-3 is present in several immune cells and is pivotal in evading the immune response by interacting with ligands. Initially, research discovered that the connection between LAG-3 and its classical ligand, major histocompatibility complex (MHC) class II, controls the suppression of T cells. Approximately ten years ago, two lectins that bind to galactose, namely galactose lectin-3 (Gal-3) and liver and lymph node sinusoidal endothelial cell C-type lectin (LSECtin), were identified as potential ligands for LAG-3. Subsequently, in 2016, α-synuclein precursor fibers (α-syn PFF) were discovered and recognized as a ligand for LAG-3, opening up new possibilities for targeting α-synuclein disease (98). This was followed by the discovery of Fibrinogen-like protein 1 (FGL1). In 2022, the T cell antigen receptor (TCR)-CD3 complex was identified as a novel ligand for LAG-3 (64). LSECtin is a type of transmembrane protein belonging to the DC-SIGN family members. It is present in various cell types, including melanoma cells, macrophages, and dendritic cells (99–102). In preclinical melanoma models, interrupting T cell immunity can be achieved through the interaction between LAG-3 and LSECtin. Interestingly, interrupted immune responses can also be reconstructed through LAG-3 antibodies (102). Gal-3 is a polysaccharide-binding protein family member and is present in many cell types, including stromal and immune cells, both intracellularly and extracellularly (103). The connection between LAG-3 and Gal-3 substantially impacts the expression of CD8 T cells. This interaction can potentially influence various aspects related to tumor spread, cell death, and resistance to chemotherapy. Gal-3 suppresses the immune response against tumors by inhibiting the activity of CD8+TIL in mice, and this effect can be reversed by removing Gal-3 (104). Another LAG-3 ligand, FGL-1, was discovered in 2019, which binds to the LAG-3 receptor with high affinity to form a higher-order LAG-3 oligomer to suppress t-cell immune responses. This oligomer plays a role in suppressing immunological responses of T-cells (77, 105). Oxymatrine decreases the production of FGL-1 by inhibiting the IL-6-driven Janus kinase/signal transducer and transcriptional activator (STAT) pathway. This enhances the sensitivity of LAG-3 immunotherapy to CD8+T cells (106). The following paragraph will comprehensively explain the recently found TCR-CD3 complex in 2022 (63). To fully understand the significance and functional role of LAG-3 ligands, it is crucial to assess their diversity. In addition, the specific part of the LAG-3 ligand in regulating inhibitory function is still unknown, and this interaction can occur in a manner that is separate, cumulative, cooperative, or opposing. Additionally, it is still being determined if specific ligands are more significant in influencing LAG-3 function than other ligands. Future mechanisms analysis will be enhanced by learning the corresponding impact of ligand interactions.

### 4.4 What are the most recent research frontiers and prospective hotspots for LAG-3 in cancer?

A visual representation of the co-occurrence of keywords in bibliometrics can serve as a network map, revealing popular research areas. Additionally, detecting burst keywords can provide new perspectives on developing and cutting-edge subjects within the field (107). We used CiteSpace to detect burst keywords between 2000 and 2023, and the top 25 keywords with the strongest citation bursts are shown in **Figure 10**. The two main keywords with citation bursts up to 2023 are “tumor microenvironment” and “PD-L1”.

The association between LAG-3 and the cancer microenvironment is nuanced. In standard physiological settings, LAG-3 exists primarily in activated T-cells, NK-cells, B-cells, plasma cells, and dendritic cells (108). However, in chronic inflammatory conditions like cancer or long-term viral infections, there is a notable increase in the expression of LAG-3 on the outer membrane of tumor-infiltrating lymphocytes, including regulatory T cells. This increase in LAG-3 contributes to the depletion of T-cells in the TME and restricts the ability of T-cells to respond against the tumor (23, 32, 109). The precise mechanism by which LAG-3 exerts its immunosuppressive effects remains incompletely elucidated. According to the available research, this mechanism involves the participation of antigen-presenting cells (APCs) in the TME and MHC II expressed on some tumor cells, along with the possible involvement of additional documented ligands.

LAG-3 ligands encompass MHC class II, Gal-3, LSECtin, FGL1, and α-syn PFF, TCR-CD3 complex (63, 74, 77, 98, 102, 104, 105, 110). Studies have demonstrated that TCR-CD3 is currently the only cis rather than trans LAG-3 ligand that can function without relying on MCH - II (63, 64). LAG-3 possesses a unique cytoplasmic tail (CT) crucial in its intracellular signaling pathway. This pathway primarily involves three motifs located in the CT: the FXXL motif, the EP motif, and the KIEELE motif (63). Among these motifs, the first two are associated with distinguishing inhibitory signals transmitted by LAG-3-pMHC class II interactions through distinct intracellular motifs. Studies have demonstrated that these three motifs are essential for LAG-3’s important roles in T-cell hybridomas (111, 112). The FXXL motif is situated at a proximal region of the membrane and possesses a serine phosphorylation site resembling CD4. This site has the potential to bind to PKC, and the activation of PKC signaling triggers the movement of LAG-3 to the cell’s outer surface.LAG-3 is speculated to significantly impact the regulation of T-cell balance by suppressing T-cell activation through the transmission of PKC signaling (63, 97).

EP motif, found at the C-terminus of the intracellular domain, is abundant in glutamate proline tandem repeat sequences (112). The interaction between CD3 and tyrosine kinase Lck, facilitated by ions, initiates the transmission of signals to T cell receptors. LAG3 molecules translocate to immunological synapses (IS) through their interaction with the TCR-CD3 complex. The EP motif can facilitate the dissociation of this complex by impeding the interaction between Zn2+ and CD3/Lck. Research has demonstrated that LAG-3 can effectively hinder its suppressive role by disrupting the link among CD4 and CD8 co-receptors and tyrosine kinase Lck (63). EP motifs can interfere with the connections between co-receptors and Lck that depend on Zn2+. Additionally, their abundance of glutamate-rich residues can disrupt the CD4-Lck complex by reducing the pH near LAG-3. Furthermore, EP motifs can also impact the signaling process after the TCR and hinder the activation of T-cells by interrupting the contacts between co-receptors and Lck. This is supported by the observation that a decrease in the binding of Lck to either co-receptor leads to a diminished capacity to phosphorylate ZAP70 (63). Furthermore, cytoplasmic structural domains lacking EP motifs are essential in facilitating the transportation of LAG-3 to the cellular membrane (97). Research revealed that inhibition mediated by the FXXL motif is more dominant than suppression mediated by the EP repeat sequence. Additionally, it was observed that the EP repeat sequence can functionally compensate for the loss of FXXL motif-dependent repression (111). The KIEELE motif, situated in the intermediate region of LAG-3’s cytoplasmic structural domain, was first discovered using truncation experiments as a crucial factor in LAG-3 signaling (112). Unfortunately, there is a lack of documentation regarding intracellular binding molecules linked to the KIEELE motif, and no research has been written on this subject (112, 113).

Below is a concise overview of the LAG-3 shedding signaling mechanism. The cleavage of LAG-3 is facilitated by two metalloproteinases, namely ADAM10 and ADAM17. These metalloproteinases individually regulate the shedding process of LAG-3, and both are influenced by TCR signaling (114). Research has demonstrated that the shedding of LAG-3 can impact the functioning of T-cells and is linked to resistance to anti-PD-1 drugs. This is supported by evidence showing that LAG-3 shedding boosts the ability of CD8+ T-cells to kill the targeted cells and promotes the effectiveness of anti-PD-1 treatment (115, 116). In general, there needs to be more knowledge regarding the precise mechanisms by which LAG-3 enhances the TCR signaling pathway and its associated effects. The mechanism by which LAG-3 uses the TCR to go to the immunological synapse (IS) has yet to be understood. In order to gain a more comprehensive comprehension of the valuable importance of LAG-3, it is necessary to have an extensive understanding of its biology, specifically its distinct intracellular cytoplasmic structural domains (known as KEEILE motifs) and its pivotal functions.

The other keyword of the burst that lasts until 2023 is “PD-L1”. LAG-3 is often found together with PD-1 in TIL. The following study provides valuable insights into the potential synergistic function of LAG-3 and PD-1 within the TME. In this research, Seng-Ryong Woo et al. (32) conducted an animal experiment and found that mice treated with anti-LAG-3/anti-PD-1 showed higher levels of IFN-γ+CD4+ and IFN-γ+CD8+ TIL and to a lesser extent, TNF-α+CD4+ or CD8+ TIL. This study demonstrates the potential efficacy of a synergistic approach involving anti-LAG-3/anti-PD-1 immunotherapy in reducing cancer progression. This is achieved by enhancing the population of effector T cells within tumors and lymph nodes. In the chronic lymphocytic choriomeningitis virus infection mouse model, Blackburn et al. showed that antigen-specific CD8+ T cells progressively lose their functionality as the infection progresses. This includes the inability to produce interleukin 2 (IL-2) in the initial phase and prolonged tumor necrosis factor (TNF) synthesis. The capacity to generate Interferon-Gamma (IFN-γ) is compromised in severe depletion. The concurrent inhibition of LAG-3 and PD-1 led to a notable augmentation in the quantity and efficacy of antigen-specific CD8 T cells while also causing a substantial reduction in viral load (23, 117). Zhi-Zhang Yang et al. (109) conducted a study on follicular lymphoma (FL) and found that CyTOF analysis revealed phenotypic heterogeneity of LAG-3+ T cells within the tumor. Flow cytometry was used to study peripheral blood samples from FL patients. It was discovered that LAG-3 was co-expressed with PD-1, and this co-expression was predominantly found on PD-1+ T cells within the tumor. In comparison to PD-1+LAG-3- cells, PD-1+LAG-3+ T cells within the tumor exhibited a diminished ability to generate cytokines, including IL-2 and IFN-γ, as well as granules such as perforin (PFN) and granzyme B (GzmB). The study additionally discovered that simultaneous inhibition of the LAG-3 and PD-1 signaling pathways yielded superior results in reversing T-cell dysfunction compared to inhibiting each path alone. This approach recovered T-cell function, enhanced cytotoxic T-cell activity, and boosted cytokine production.

The majority of the studies above primarily investigated T cells and primarily used Flow cytometry. However, Jani Huuhtanen et al. (118) took a different approach by utilizing single-cell RNA and T-cell receptor sequencing (scRNA+TCRαβ-Seq) alongside other multi-omics techniques to analyze samples of peripheral blood from 40 melanoma patients who received therapy with a combination of relatlimab and nivolumab. Their findings revealed that LAG-3 was expressed in CD8+ T cells and exhibited high expression in NK cells and Tregs among melanoma patients. The level of expression of LAG-3 showed a strong correlation with the degranulation response of NK cells. Following treatment with anti-PD-1 + anti-LAG-3, NK cells exhibited degranulation and released cytokines, while CD8+ T cells underwent proliferation. The research additionally discovered that the combination of therapies enhanced the cytotoxic characteristics of NK cells stimulated antigen-restricted T cells, and modified the expression profile of Tregs. Further investigation is required to understand the mechanisms behind the synergistic effects of LAG-3 and PD-1 and determine why combination therapies are effective.

Currently, medicines targeting LAG-3 can be classified into three groups: monoclonal antibodies targeting LAG-3, LAG-3 protein fusions, and antibodies targeting both LAG-3 and another molecule simultaneously (119). LAG-3 monoclonal antibodies (mAb) can be classified into humanized IgG4 antibodies and the new Fc-engineered immunoglobulin G1κ monoclonal antibody INCAGN02385. Between them, relatlimab (BMS-986016) is the most prominent example of LAG-3 mAb (120, 121). Based on current investigations, using only anti-LAG-3 as a form of therapy may not be the best option. Research has discovered that LAG-3 and PD-1 are simultaneously present in CD8+ T-cells infiltrating tumors. This finding was confirmed by testing on mice, where integrating anti-LAG-3 and anti-PD-L1 mAbs indicated an even more significant therapeutic effect than using either antibody alone (32, 122–124).

In 2022, the U.S. Food and Drug Administration (FDA) approved the drug combination of relatlimab and nivolumab, known as Opdualag, to treat unresectable or metastatic melanoma. This decision was based on the positive outcomes observed in the RELATITY-047 trial (125, 126). This advancement marks a crucial turning point as LAG-3 becomes the third checkpoint inhibitor to exhibit efficiency in clinical targeting. The median progression-free survival with Opdualag was 10.1 months (95% CI, 6.4-15.7), while for nivolumab it was 4.6 months (95% CI, 3.4-5.6). Furthermore, compared to nivolumab, Opdualag had superior effectiveness, as evidenced by a 12-month progression-free survival rate (PFS) of 47.7% instead of 36%. Opdualag demonstrated favorable efficacy and a reduced incidence of adverse effects in comparison to combo therapy. The reaction outcome of Opdualag exhibited a comparable pattern to that of the ipilimumab/nivolumab combination, which consists of anti-CTLA4 and anti-PD-1 drugs. Opdualag achieved a 12-month PFS Rate of 47.7%, whereas ipilimumab/nivolumab had a PFS rate of 49%. Nevertheless, ipilimumab/nivolumab resulted in a much higher proportion of patients (59%) experiencing notable adverse events, in contrast to only 18.9% with Opdualag (127, 128). Ascierto et al. conducted a study to assess the effectiveness of nivolumab and relatlimab in treating resectable stage III/IV melanoma and visceral non-pulmonary metastases before surgery. The study revealed that the concurrent administration of anti-LAG-3/anti-PD-1 therapy benefits patients who did not respond to initial anti-PD-1/PD-L1 therapy (129). Hence, considering its exceptional safety, cost-efficiency, and high probability of patient survival, it is probable that relatlimab/nivolumab will emerge as the favored first-line treatment for advanced melanoma and neoadjuvant therapy.

Eftilagimod alpha (IMP321) is the sole soluble LAG-3 protein capable of stimulating APCs and subsequently activating CD8 T cells. If used by itself, its clinical efficacy is limited. However, research has demonstrated that IMP321 works efficiently with cytotoxic chemotherapy and vaccine-based methods. Specifically, IMP321 with gemcitabine, which is for individuals with advanced pancreatic cancer, has shown promising results in terms of tolerance and security (130). Following the findings of the TACTI-002 trial, the FDA has given Eftilagimod alpha, when combined with pembrolizumab, a rapid-track designation as a first-line therapy for individuals with stage III B/IV NSCLC who have a PD-L1 TPS score of less than 1%. This study assessed the efficacy of Eftilagimod alpha (IMP321) in conjunction with pembrolizumab for treating people who have previously untreated inoperable or metastatic NSCLC, recurring PD-(L)1 resistant NSCLC, or recurring or metastatic HNSCC. The median duration of response (DOR) in the initial therapy for those with NSCLC was 21.6 months, whereas the median PFS was 6.6 months (131). The objective response rate (ORR) for a combination of treatments in the second-line therapy for individuals suffering from PD-L1-nonselective HNSCC was 39% (132). Tebotelimab (MGD013) is a tetravalent DART molecule meant for studies. It contains Fc and is intended to attach to PD-1 and LAG-3 to preserve, maintain, or regain the function of T cells that have been exhausted. The combination of Tebotelimab and Margetuximab demonstrates promising clinical efficacy in patients with advanced HER2-positive tumors (133). ABL501 is a type of bispecific antibody that explicitly targets LAG-3 and PD-L1. It can stimulate the activation of DC and facilitate the attachment of tumor cells to T cells. This antibody is utilized in regulating the immune cell responses against cancers. ABL501, a potent inhibitor of the LAG-3 and PD-L1 pathways, significantly augments the stimulation of CD4+ and CD8+ T cells to a greater extent than an all-encompassing combination of anti-LAG-3 and anti-PD-L1. This enhanced impact can result in a decrease in immunosuppression due to regulatory T cells (124).

Prior research has primarily focused on investigating the integrated impact of LAG-3 and PD-1 inhibitors. Additional investigation is required to comprehensively comprehend the mechanism underlying the synergistic therapy combining LAG-3 and PD-1. This investigation is of utmost importance as it has the potential to influence the optimal selection of a novel combination therapy. The United States FDA has approved and Drug Administration two LAG-3 medications with PD-1 inhibitors for treating individuals with solid tumors. Patients diagnosed with metastatic melanoma with no previous medical intervention are administered a combination treatment consisting of relatlimab+nivolumab and nivolumab+ipilimumab, which involves the use of an anti-CTLA-4 monoclonal antibody. The two medicines have no significant difference in the 12-month PFS rate. However, the differentiation between them lies in their negative impacts. Therefore, might utilizing three ICIs specifically targeting PD-1/PD-L1, CTLA-4, and LAG-3 provide a better alternative for cancer patients? Currently, phase II clinical research is underway to evaluate the safety and efficacy of combination therapy utilizing ipilimumab, nivolumab, and relatlimab for the treatment of advanced stage III or IV melanoma that is not amenable to surgical removal (NCT0548007). Which additional therapeutic approaches should be combined with LAG-3 treatment? With scientists recognizing other inhibitory receptors such as TIGIT, TIM-3, CD160, and 2B4, it is pertinent to contemplate the efficacy of LAG-3 in binding to these receptors. In addition to immunotherapy, malignancies can be treated utilizing various modalities such as chemotherapy, radiation, cellular therapy, and photodynamic treatment. Can the combination of LAG-3 therapy with these medicines result in surprising results? To fully capitalize on the conceivable advantages of this treatment approach, it is necessary to make further endeavors to improve efficacy and safety while reducing the frequency of immune-related adverse consequences in various disease populations for the different implementations of LAG-3 targeted treatment.

### 4.5 Strengths and limitations

This work is the first known attempt to perform a bibliometric analysis of cancer research related to LAG-3 based on the available information. The primary objectives of this study are to ascertain the collaborative relationships among authors, countries, and institutions and to elucidate the evolutionary trajectory, current hotspots, and prospective directions within this field. Nevertheless, this study has limitations, which can be attributed to many factors. This study exclusively utilized journal papers from the WoSCC database, explicitly focusing on publications indexed in the SSCI and SCIE databases. The dataset comprised 1841 articles published after the year 2000. Notably, other databases, such as PubMed and Scopus, were not included in the analysis. Guarantee the integrity and reliability of the data, this study omitted papers published before 2000. However, this decision resulted in a drawback of the data analysis due to the absence of information from that period.

Furthermore, most of the studies incorporated in our analysis were performed in English. Selection bias is possible since we may have overlooked relevant publications published in other languages (134). Additionally, while using professional tools for bibliometric analysis is considered objective, it is essential to acknowledge that researchers’ subjective perspectives can influence the interpretation and comprehension of the area. This study endeavors to reduce the limitations of depending solely on the opinion of a single researcher by facilitating thorough discussions among numerous writers.

## 5 Conclusions

The quantity of academic publications about LAG-3 research in the domain of oncology has exhibited a notable surge in growth since 2013. The top three journals regarding productivity are Frontiers in Immunology, Journal of Immunology, and Cancers. The Journal of Immunology, Journal of Experimental Medicine, and Immunity are widely acknowledged as the three most commonly co-cited publications within the field. The average citation count per publication is significantly higher for four authors affiliated with the United States. Additionally, authors from the United States exhibit a strong propensity towards international collaboration, whereas Triebel, F, hailing from the UK, participates in comparatively limited collaboration with authors from other countries. The United States occupies a prominent position in the field of study about this specific subject, making a substantial contribution of 47.51% of the overall academic publications. The countries of the United States, China, and Germany, collectively contributing to 69.69% of the overall quantity of papers, have played an enormous part in advancing the progress of LAG-3 research. The research project on LAG-3 in the realm of oncology in China launched at a somewhat delayed stage, with a greater emphasis on collaboration between Chinese institutions and domestic counterparts, while exhibiting lesser cooperation with international organizations. Numerous challenges remain to be resolved in the forthcoming period. Our current knowledge of LAG-3 is inadequate at the epigenetic change, transcriptional, and post-translational levels and our comprehension of its interactions with ligands is still in its nascent phase. Furthermore, by thoroughly investigating the TCR- CD3 complex, the only cis ligand of LAG-3, we can get significant insights into the signal transduction mechanism involving LAG-3. Hence, it is imperative to boost the investigation of the binding locations of LAG-3 and TCR-D3 complexes, along with the LAG-3 domain and residues that facilitate interactions with TCR-CD3 complexes. Ultimately, it is necessary to investigate novel amalgamations of LAG-3 therapy, which have the potential to result in improved therapeutic strategies and curative interventions for diverse tumor types.

## Data availability statement

The original contributions presented in the study are included in the article/**Supplementary Material**. Further inquiries can be directed to the corresponding author.

## Author contributions

JW: Formal Analysis, Investigation, Methodology, Visualization, Writing – original draft. SW: Investigation, Writing – original draft. YZ: Investigation, Writing – original draft. WZ: Conceptualization, Investigation, Supervision, Writing – review & editing.
