# Supplementary material for: Bibliometric analysis of evolutionary trajectory and prospective directions of LAG-3 in cancer
Source: Front Immunol. 2024 Feb 8;15:1329775. doi: 10.3389/fimmu.2024.1329775 (PMC10881671; doi:10.3389/fimmu.2024.1329775)
Supplement: Supplementary file 1 [file DataSheet_1.docx]

Supplementary Material

[**Supplementary material 1**](Manuscript.docx)

Figures 1A and 1C are based on research results from Delft University of Technology, The Netherlands.

The reference material website is as follows:

<https://www.tudelft.nl/en/library/research-analytics/case-21-tu-delft-top-collaborators-2>

The sources of geographic data packets are as follows:

<http://thematicmapping.org/downloads/world_borders.php>，

In this study, we mainly use "map.txt" and "net.txt" in the geodata download package, and the following are the steps for FIGURE 1A mapping (FIGURE 1C and FIGURE 1A steps are the same):

STEP 1: We need to do a synonym merge before we can do the country collaboration network map, the details of the country synonym merge are explained in the article.

STEP 2: Set 12 as the minimum threshold and build a national cooperation network map. Afterwards, we click save and name the saved "VOSviewer map file" and "VOS network file" as "map1. txt" and "net1. txt".


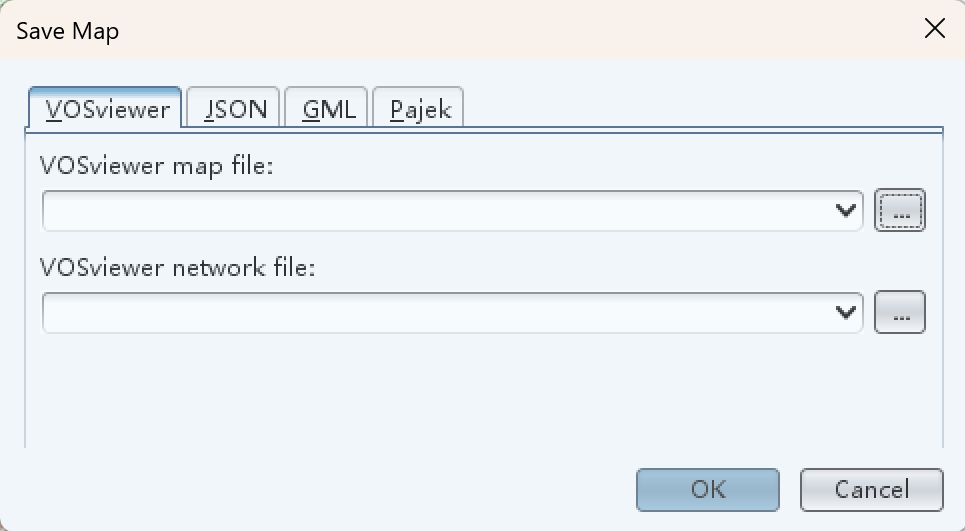


STEP 3: Copy the contents of "map1.txt" to a new EXCEL file named "map1.xlsx".


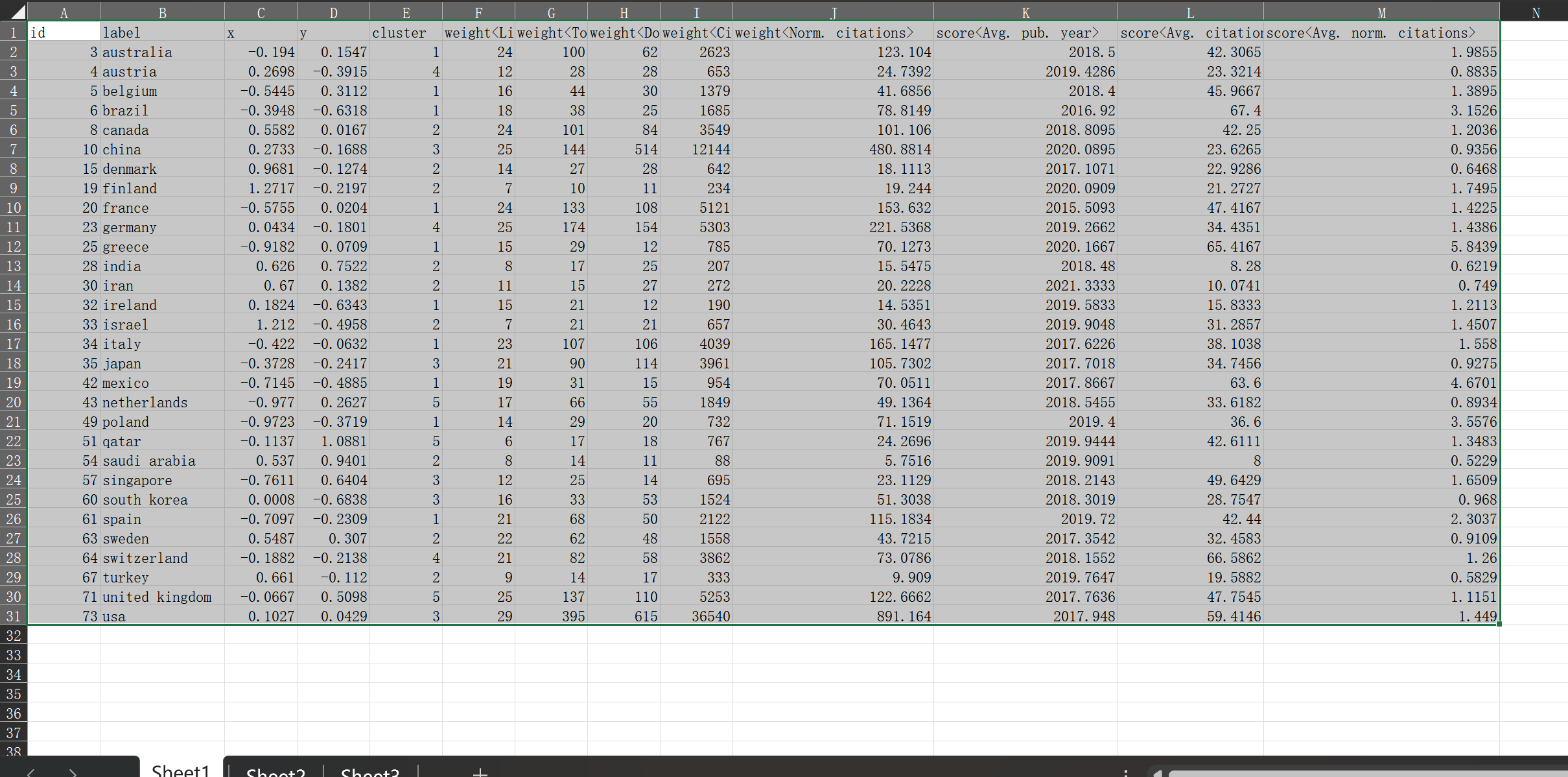


STEP 4: Retrieve the latitude and longitude of each country in your browser, replacing the values in columns X and Y in the original table, with column X as the horizontal coordinate and column Y as the vertical coordinate.

STEP 5: Copy the information related to "map. txt" from the geodata download package into "map1. xlsx" and delete several rows of data values in the "map1. xlsx" file that are related to the country data in "map. txt" (the country data in "map1. txt" was used in this study, as shown in the figure below).

5.1 The contents of map.txt are as follows:


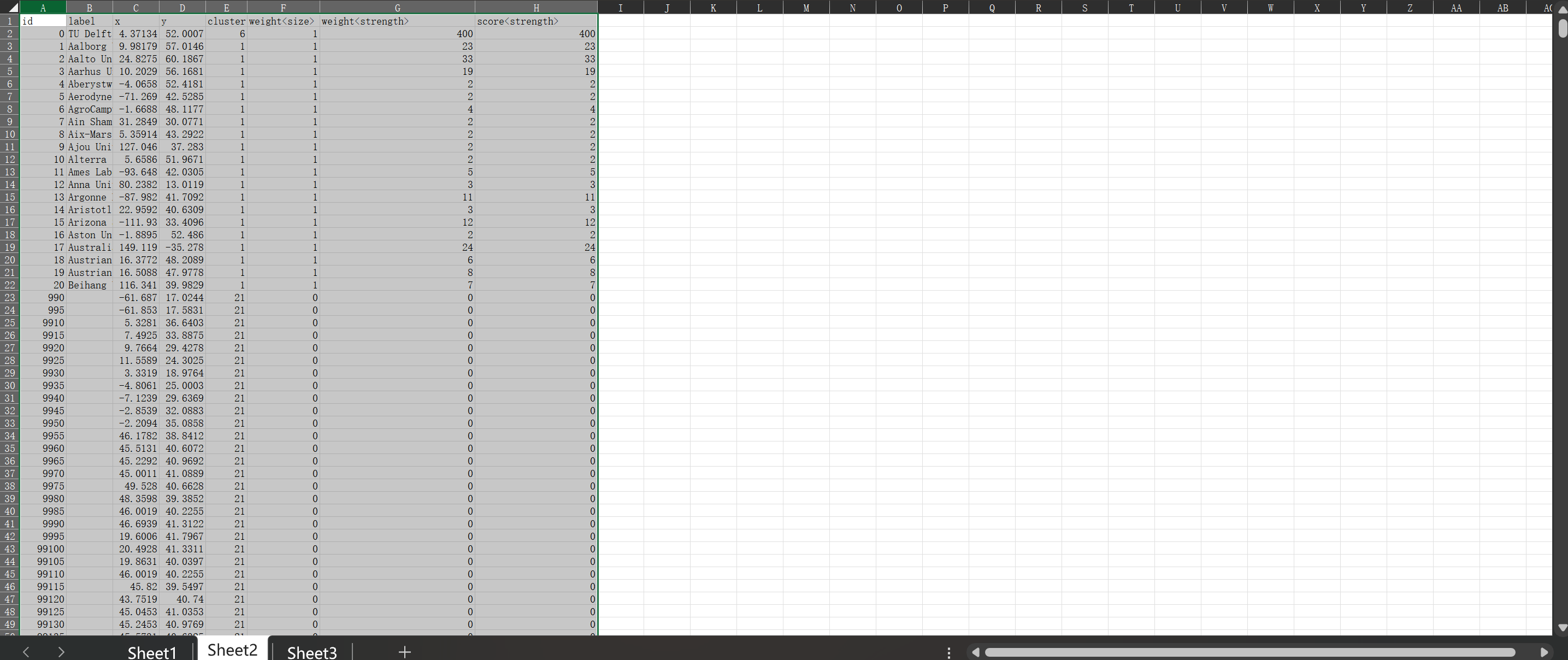


5.2 Copy the information from "map.txt" to "map1.xlsx" in the downloaded geographic data package. The content is as follows:


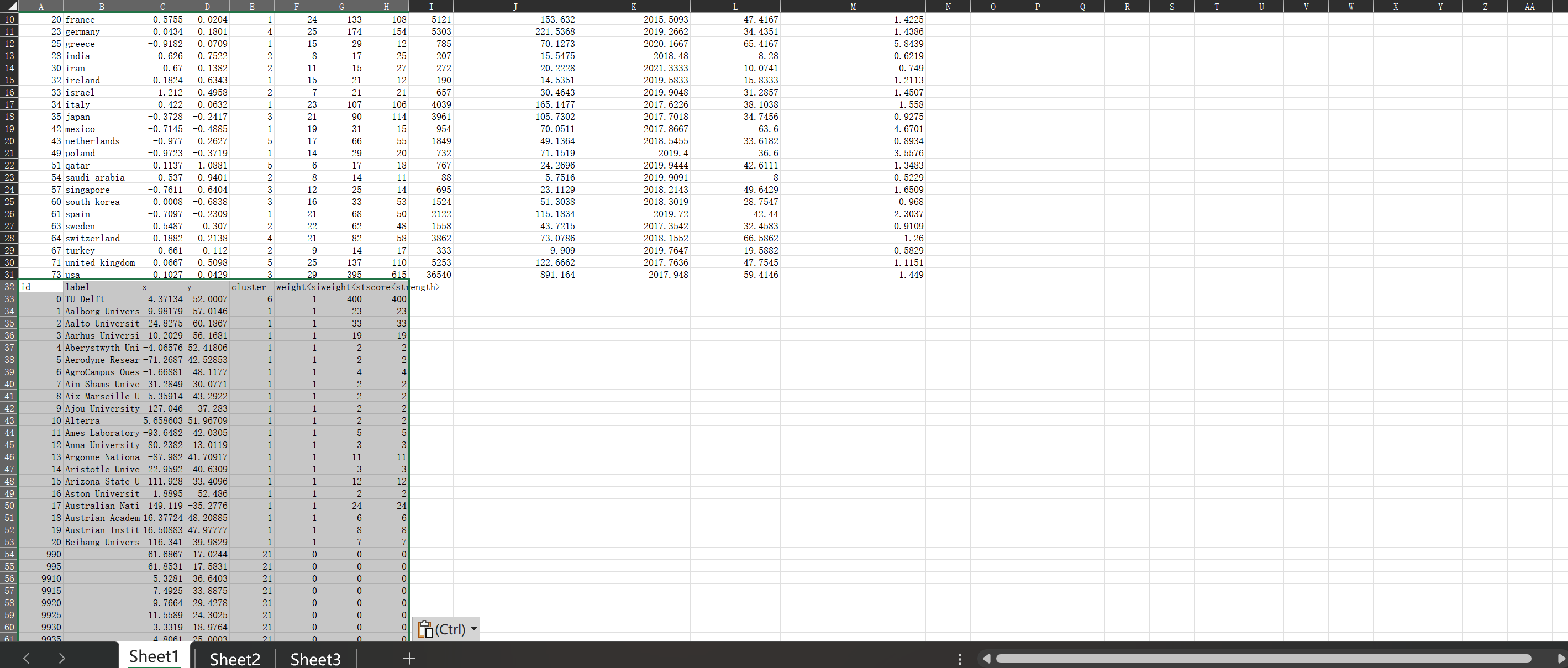


5.3 Supplementing the blanks with zeros, the final display results are as follows:


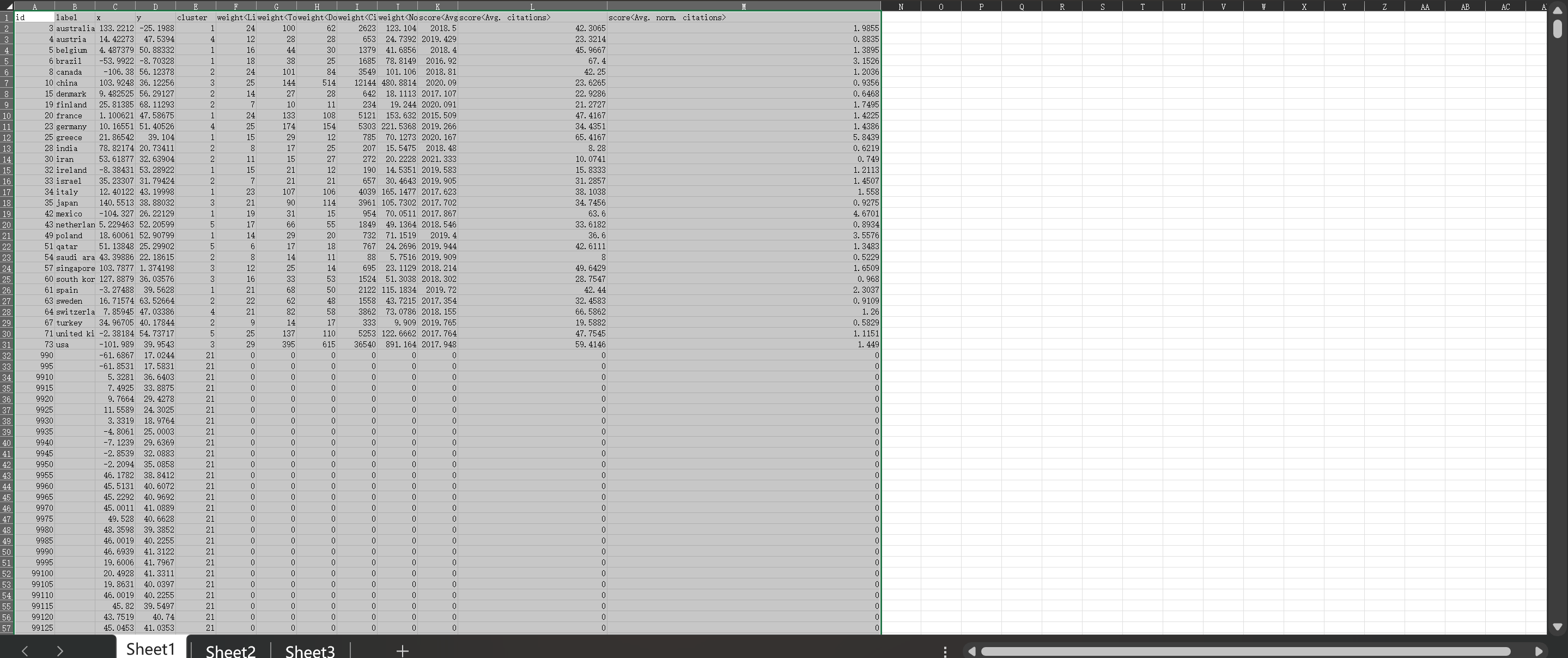


STEP 6: Name "map1.xlsx" as "mapnew.xlsx", convert the format to "mapnew.txt".

STEP 7: Open "mapnew.txt" and "net1.txt" in VOSviewer.

STEP 8: Adjusting background and node colours.

Step9: FIGURE 1A production completed.

**Supplementary material 2**

The creation of Figure 2 requires the use of the software VOSviewer and Pajek.

STEP 1：Download Pajek with the green logo from the official website at the following address.

<http://mrvar.fdv.uni-lj.si/pajek/>


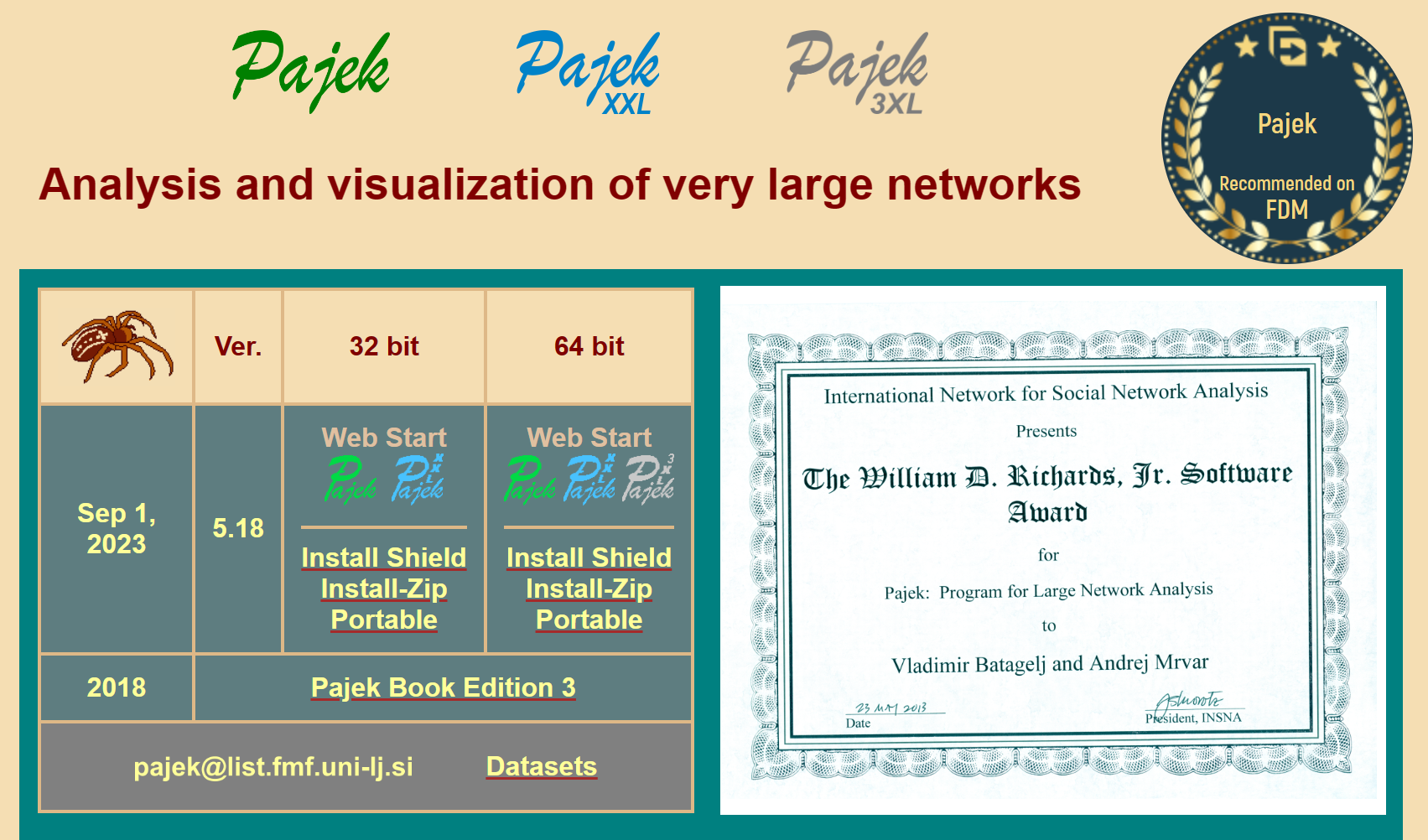


STEP 2: Set the minimum threshold to 14 to get the keyword visualisation map, click save, name the saved "VOSviewer map file" and "VOS network file" as "map.txt", "net.txt".


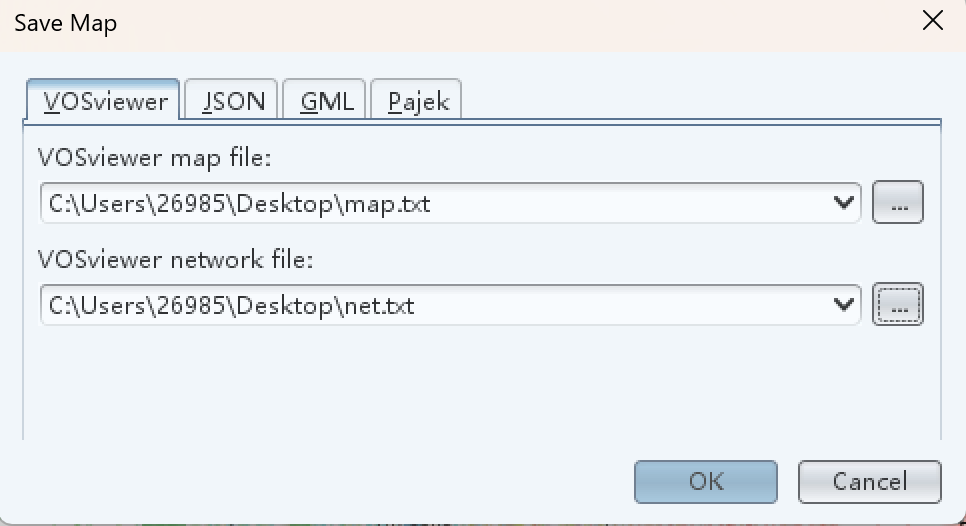


STEP 3: Save the Pajek files as "1.net" "1.clu" "1.vec".


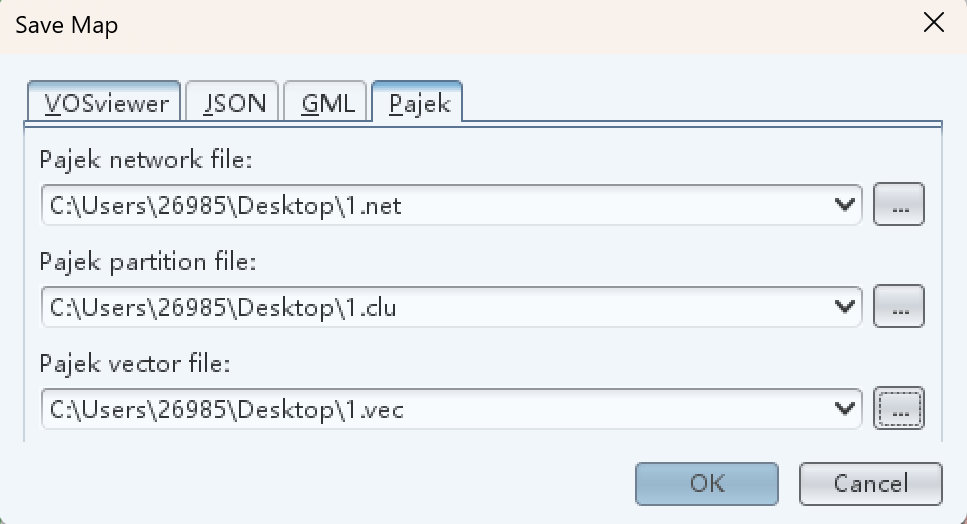


STEP 4: Import the file from step 3 into Pajek.


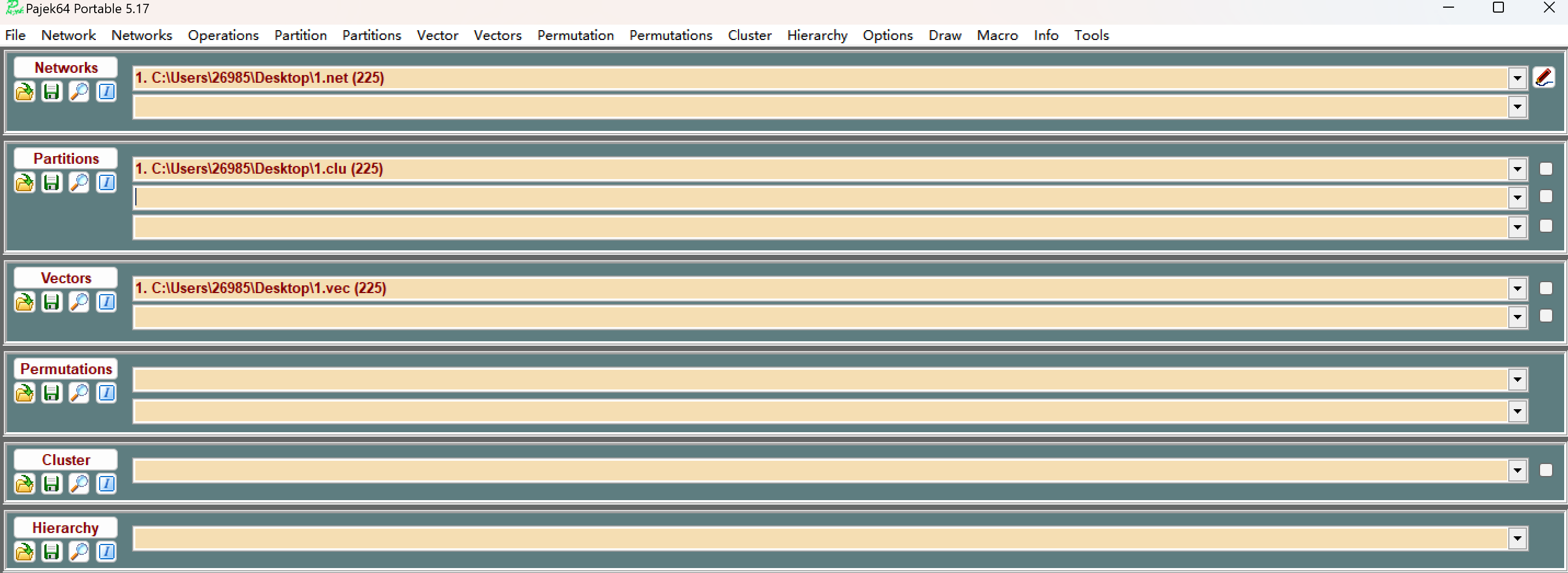


STEP 5: Click on "draw" and select “Network + first Partition+ First Vector”


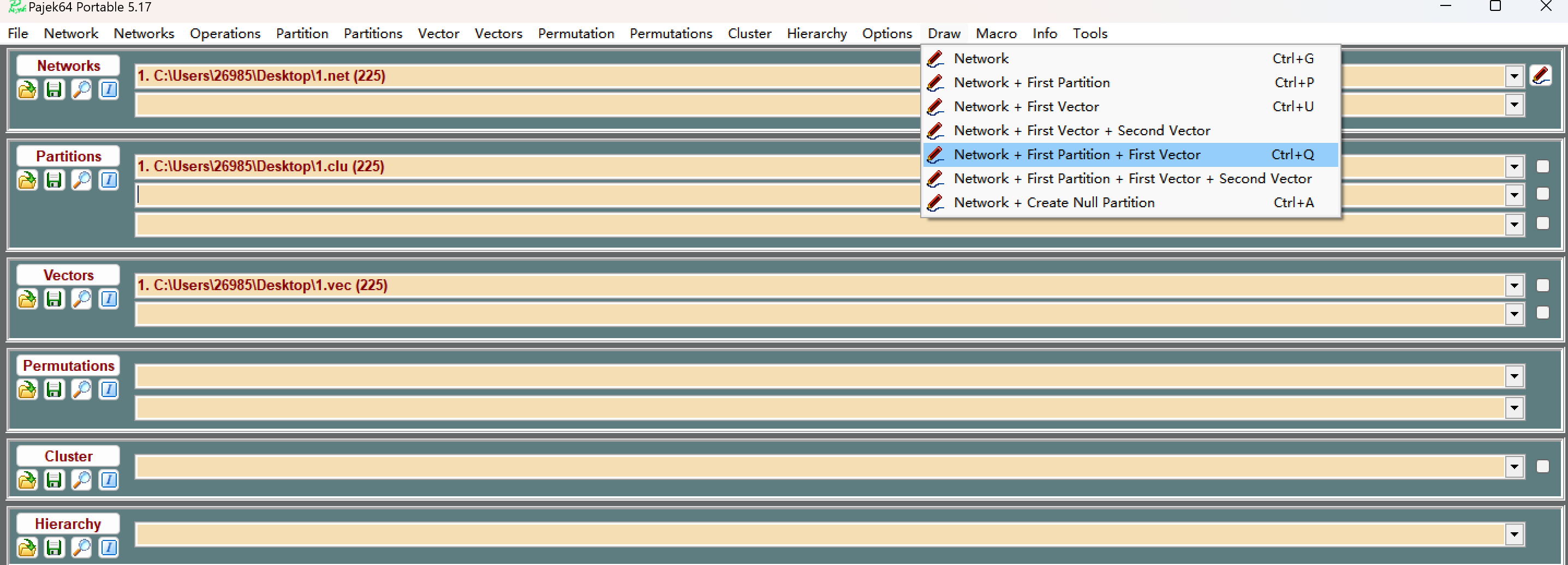


STEP 6: Select “layout”-Energy-kamada kawai-Optimize inside Clusters only


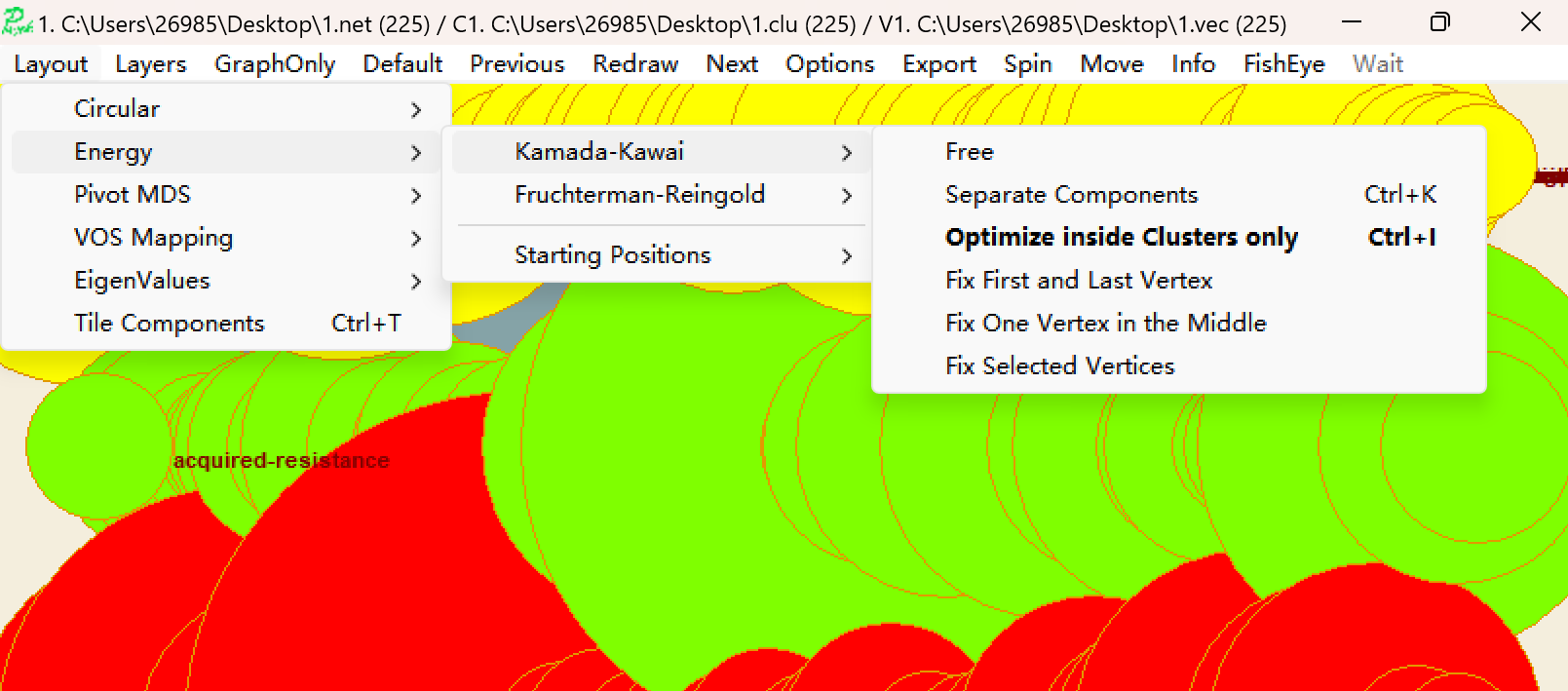


STEP 7: Select “Layers ---In y Direction”


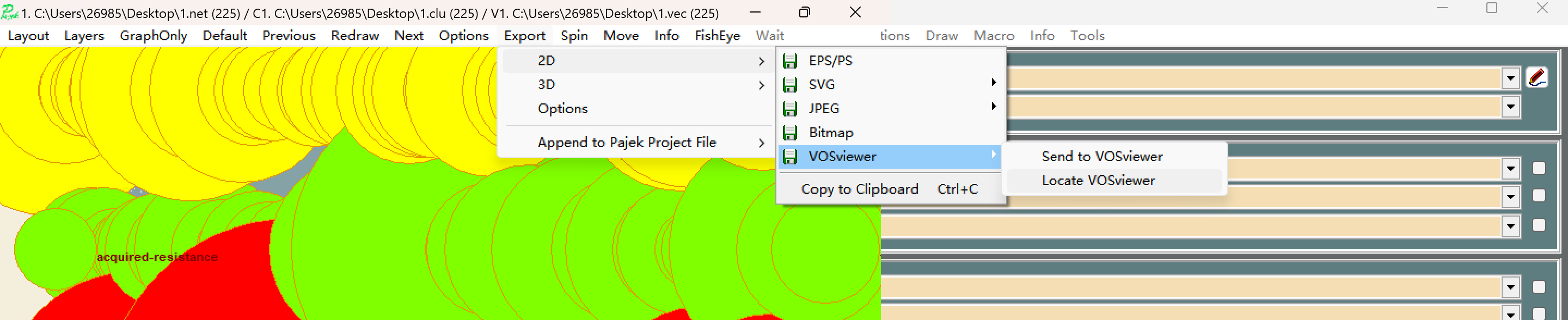


STEP 8: Select “Export ---2D---VOSviewer---send to VOSviewer”（(Note: For the first export, you need to select “Locate VOSviewer” – “Generate a new keyword visualization map.”）

STEP 9: Save a new save file named "map1.txt", "net1.txt".


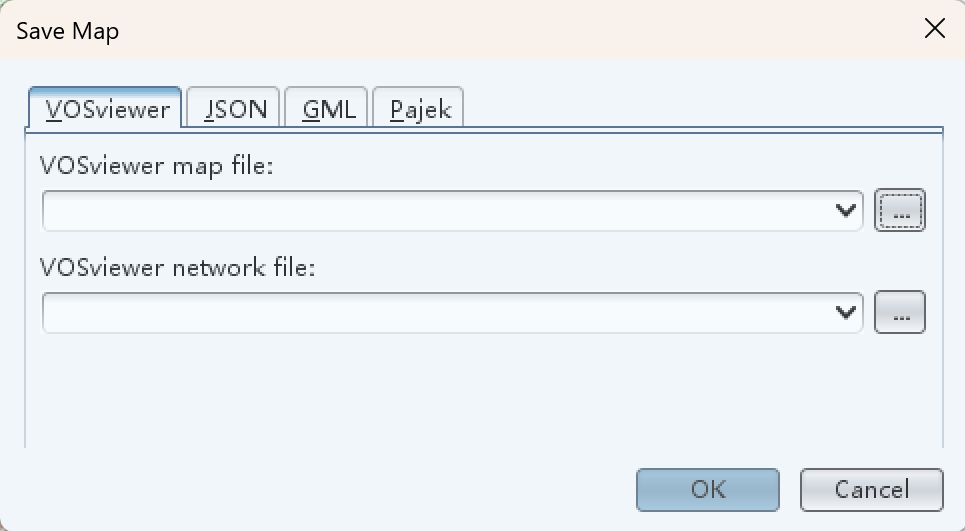


STEP10：The following is the difference between "map.xlsx" and "map1.xlsx" files, so we have to add the "I, J, K" columns in "map.xlsx" to "map1.xlsx", "I, J", K "contains VOSviewer's time information.

The information in "map.xlsx" is as follows:
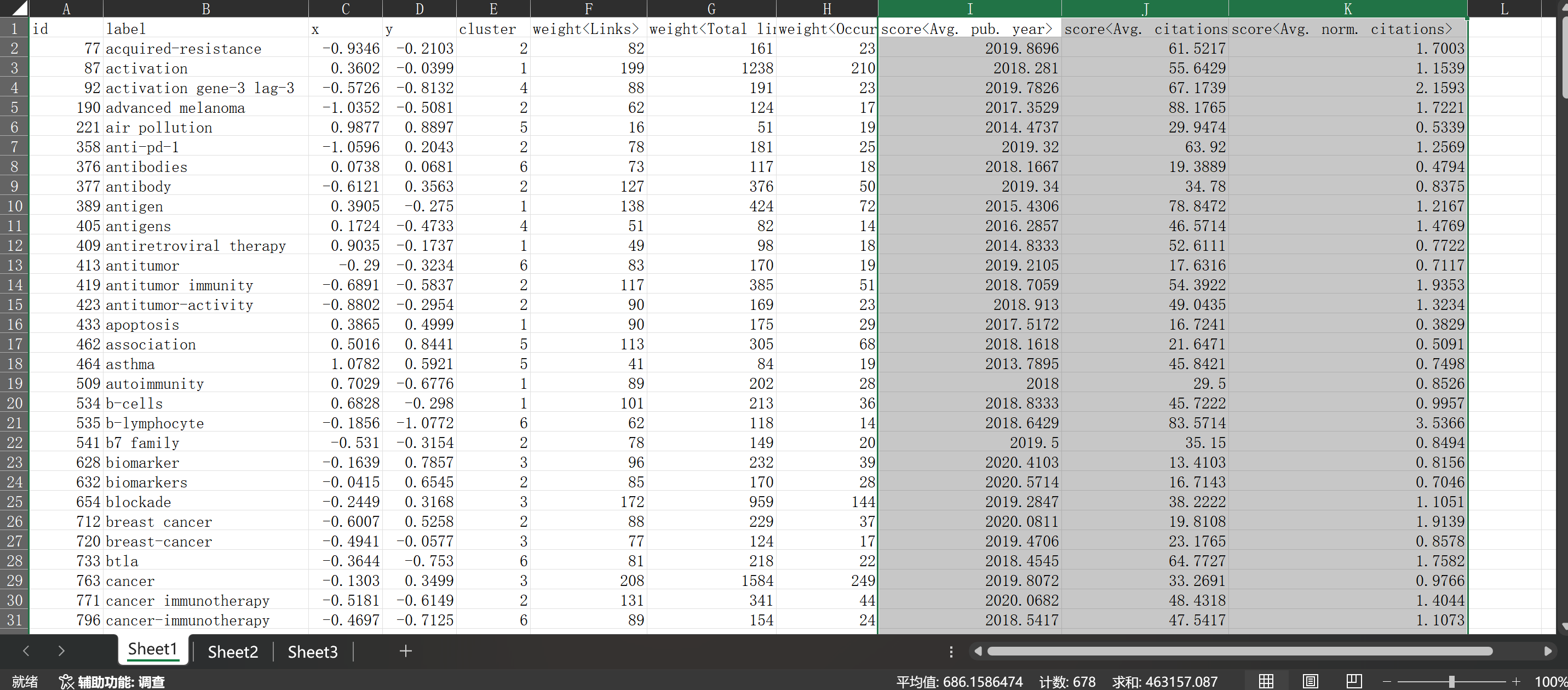


The information in "map1.xlsx" is as follows:


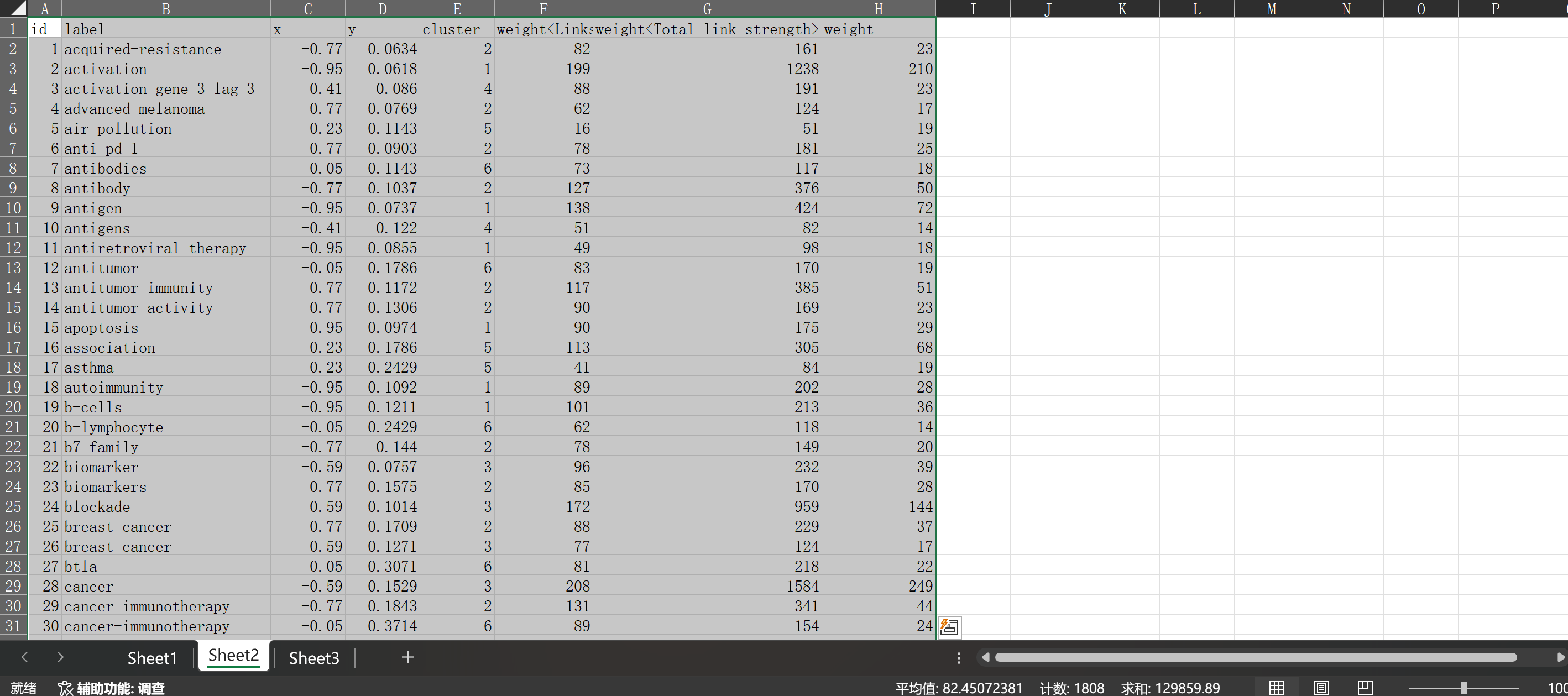


STEP 11: Name the file "map1.xlsx" after adding the data information as "mapnew.xlsx" and convert the format to "mapnew.txt".

STEP 12: Open "mapnew.txt" and "net1.txt" in VOSviewer, FIGURE 2 is finished.
